# Polypyrrole/Graphene/WO_3_ Ternary Nanocomposite Sensor Arrays for 3D Ammonia Leakage Reconstruction via Rolling Horizon Data-Assimilated PINNs

**DOI:** 10.3390/polym18172149

**Published:** 2026-09-02

**Authors:** Haodong Niu, Yunbo Shi, Kuo Zhao, Jinzhou Liu, Xiaohui Yang, Canda Zheng

**Affiliations:** 1 School of Measurement and Control Technology and Communication Engineering, Harbin University of Science and Technology, Harbin 150080, China; 2010600001@stu.hrbust.edu.cn (H.N.); iridescen7@163.com (K.Z.); liujinzhou0709@outlook.com (J.L.); 420610159@stu.hrbust.edu.cn (X.Y.); 2410603075@stu.hrbust.edu.cn (C.Z.); 2 China Higher Educational Key Laboratory for Measuring & Control Technology and Instrumentations of Heilongjiang Province, School of Measurement-Control Technology and Communications Engineering, Harbin University of Science and Technology, Harbin 150080, China

**Keywords:** PPy/Graphene/WO_3_ nanocomposite, ammonia detection, physics-informed neural networks (PINNs), 3D diffusion reconstruction

## Abstract

To address the limitations of conventional discrete ammonia-monitoring schemes, this study proposes a closed-loop sensing–inference architecture that integrates a distributed chemical-sensing network with a physics-informed neural network (PINN) to achieve high-fidelity three-dimensional dynamic reconstruction of gas diffusion in confined environments. A distributed sensor array based on a PPy/Graphene/WO_3_ ternary nanocomposite provides real-time wireless monitoring and reliable observational data streams owing to its sub-ppm detection limit and high signal-to-noise ratio. Fick’s second law of diffusion and Neumann no-flux boundary conditions are embedded in a mesh-free PINN, and rolling horizon data assimilation (RHDA) is introduced to dynamically fine-tune the network weights with high-frequency real-time observations, thereby establishing a real-time closed-loop correction between theoretical inference and the monitored environment. The proposed method achieves accurate three-dimensional concentration-field reconstruction under steady-state conditions, markedly suppresses errors and maintains robustness under unknown abrupt concentration disturbances, and mitigates the temporal divergence of conventional data-driven models during long-term purely physical extrapolation without data support. Thus, the framework combines hardware sensitivity, computational efficiency, and macroscopic physical generalization.

## 1. Introduction

Ammonia (NH_3_) is a highly irritating and corrosive alkaline gas, the leakage of which not only causes environmental pollution but also poses a direct threat to human health [1,2]. Previous studies have shown that acute exposure to high concentrations of NH_3_ can induce severe respiratory damage, such as laryngitis, bronchitis, and emphysema. Furthermore, even chronic exposure to NH_3_ levels of ~200 ppm may cause persistent irritation of the eyes, nose, throat, and respiratory tract in both children and adults, increasing the risk of acute asthma exacerbations and other pulmonary diseases [3,4]. Concurrently, NH_3_ is a significant volatile byproduct of nitrogen metabolism in the human body, with abnormal NH_3_ concentrations in exhaled breath or biological fluids being closely linked to hepatic diseases, *Helicobacter pylori*-related conditions, and renal dysfunction, thereby rendering it a critical biomarker for medical diagnosis [4,5,6]. Furthermore, in confined environments, such as hazardous chemical warehouses and storage rooms, nitrogen-containing chemicals (e.g., ammonium nitrate) may undergo slow decomposition and release trace amounts of NH_3_ under adverse storage conditions. Rapid detection and warning of indoor NH_3_ can therefore significantly mitigate the risks of explosion and poisoning accidents, ensuring the safety of personnel [7,8]. In complex environments, such as confined spaces, NH_3_ diffusion is governed by multiple factors, including thermal buoyancy, physical wall constraints, and local convection, resulting in highly nonlinear and transient evolution characteristics of the concentration field [9,10,11]. Consequently, the development of accurate, real-time, and effective NH_3_ detection technologies is of great significance for environmental pollution control, industrial safety, and auxiliary medical diagnosis. In the context of confined spaces, the development of monitoring technologies capable of achieving the real-time, high-fidelity reconstruction of three-dimensional (3D) continuous concentration fields possesses profound scientific value and practical significance for early dynamic disaster monitoring and precise emergency rescue decision-making.

Although current metal oxide semiconductor (MOS) sensors exhibit high sensitivity, they typically require high operating temperatures exceeding 200 °C, rendering it difficult to meet the demands of large-scale distributed, low-power monitoring [12]. Polypyrrole (PPy) is considered a candidate material for the construction of low-power NH_3_ sensors as it can be conveniently synthesized through chemical oxidative polymerization or electrochemical polymerization. Additionally, the conductivity of PPy can be tuned through dopants, acidity, and polymerization conditions, and the material can operate at significantly lower temperatures [13]. Despite these advantages, pure PPy still exhibits limitations such as slow response and recovery rates, together with insufficient sensitivity and long-term stability. Studies have shown that morphological control and doping with functional fillers are effective strategies for enhancing its gas-sensing performance [14,15]. Among these fillers, graphene, a typical two-dimensional (2D) carbon nanomaterial, possesses a high specific surface area, high carrier mobility, low noise, and room-temperature responsiveness. Its introduction as a filler can generate continuous conductive channels and reduce device power consumption. Notably, by loading PPy nanoparticles onto graphene, Casanova-Chafer et al. achieved trace NH_3_ detection at room temperature, with the device exhibiting good repeatability (relative error ≈ 0.7%), a low detection limit of 491 ppb, and favorable long-term stability [16]. Although carbon-based nanomaterials can enhance the charge transport and adsorption interfaces of conducting polymers, systems relying on a single carbon-based modification strategy still exhibit limitations in response intensity and selectivity. In recent years, composites of PPy with n-type metal oxides (e.g., WO_3_) have attracted growing attention due to their ability to form a p-n heterojunction and introduce more abundant surface active sites. In this context, Filipescu et al. fabricated WO_3_/PPy nanocomposites using a laser deposition technique and achieved room-temperature NH_3_ detection, indicating that the heterointerface between WO_3_ and PPy enhances the response to NH_3_ [17]. Therefore, the integration of zero-dimensional (0D) PPy, 2D graphene, and one-dimensional (1D) WO_3_ into a ternary composite is expected to simultaneously leverage the room-temperature conductive response of PPy, the efficient charge transport properties of graphene, and the catalytic and heterojunction modulation effects of WO_3_. This approach should therefore provide a solid material foundation for the construction of highly sensitive, low-power NH_3_ sensing nodes suitable for distributed monitoring.

ZnO, SnO_2_, TiO_2_, and MoS_2_ are also viable NH_3_-sensitive fillers, and suitably modified or heterostructured forms can operate at room temperature. For the present room-temperature, low-power design, material selection was based primarily on the complementary conductive-network and heterojunction roles of graphene and WO_3_. Graphene was selected as the two-dimensional conductive component because it forms a high-surface-area, continuous charge-transport network and has been validated in PPy-based room-temperature NH_3_ sensing [16]. Hydrothermally prepared WO_3_ nanorods were selected as the one-dimensional n-type oxide because they provide adsorption and catalytic surface sites and form an interface with p-type PPy that has been reported to enhance the room-temperature NH_3_ response [17]. By comparison, ZnO, SnO_2_, TiO_2_, and MoS_2_ require separate optimization of defect chemistry, dispersion, interfacial energy levels, and activation conditions [12]. The composition was optimized sequentially under 50 ppm NH_3_ at room temperature: varying the graphene content from 0 to 2 wt% produced a maximum response of 31.31% at 1.5 wt%; after fixing this loading, varying the WO_3_ content from 2 to 8 wt% produced a maximum response of 52.49% at 6 wt%, which was 1.68 times that of PPy/Gr3. Accordingly, PPy/Gr3/WO_3_-3 was the optimum composition within the investigated ranges. Studies of graphene/polymer nanocomposites and graphene-enabled thermal management further indicate that stable dispersion, continuous conductive networks, and interfacial transport are central to the functional performance of graphene-containing systems [18,19].

Computational fluid dynamics (CFD) simulations provide high-fidelity physical fields for constructing gas-diffusion models; however, the high cost of solving fully coupled Navier–Stokes equations limits their suitability for online early-warning applications [20,21]. Data-driven deep-learning models have increasingly been applied to flow-field prediction, but open-loop models without physical constraints are prone to temporal divergence under unknown environmental disturbances [22]. In complex environments such as confined warehouses, discrete sensor arrays provide only local concentration measurements and cannot directly represent the global three-dimensional spatiotemporal evolution of an NH_3_ leak [23,24,25]. Cai et al. showed that PINNs can reconstruct flow fields and invert parameters from sparse observations by embedding governing equations such as the Navier–Stokes equations in the loss function; nevertheless, long-term extrapolation remains susceptible to numerical divergence during complex transient processes involving strong convection or high Reynolds numbers [26]. A related CNT-TiO_2_ electronic-nose study combined heterogeneous nanostructured sensors with PCA/SVM analysis and achieved 97.5% classification accuracy for room-temperature VOC discrimination, providing a relevant reference for integrating sensing and artificial intelligence [27].

To overcome the limitations of conventional gas-sensing systems that provide only discrete measurements and employ reconstruction models independently of the sensing hardware, this study proposes a closed-loop sensing–inference framework for dynamic NH_3_ monitoring in confined spaces. At the sensing level, 0D PPy nanospheres, 1D WO_3_ nanorods, and 2D graphene nanosheets are integrated into a three-dimensional heterogeneous conductive network that combines the room-temperature response of PPy, the efficient charge transport of graphene, and the catalytic and heterojunction effects of WO_3_ to provide sensitive, low-noise NH_3_ observations. At the system level, a customized 27-channel array provides spatially distributed concentration measurements. At the reconstruction level, Fick’s diffusion equation and Neumann boundary conditions are embedded in a mesh-free PINN, while RHDA periodically incorporates distributed observations to update the model and suppress temporal error accumulation. The principal innovation is the closed-loop coupling of the room-temperature PPy/Graphene/WO_3_ sensing layer with RHDA-PINN, which converts discrete observations into a continuous three-dimensional NH_3_ concentration field and is evaluated in terms of static accuracy, robustness to abrupt source disturbances, and long-term extrapolation.

## 2. Sensor Fabrication and Sensing Performance

This section focuses on the physical sensing layer of the proposed architecture, including the preparation, structural analysis, and room-temperature NH_3_-sensing performance of the PPy/Graphene/WO_3_ composite. The fabricated sensing material and acquisition hardware constitute the experimentally characterized front-end basis, while the measured response magnitude, dynamic characteristics, selectivity, environmental dependence, and stability define the practical sensing requirements.

### 2.1. Materials and Preparation

Pyrrole (C_4_H_5_N; AR grade, Macklin, Shanghai, China), ammonium persulfate ((NH_4_)_2_S_2_O_8_; AR grade, Fuchen Reagent Factory, Tianjin, China), Na_2_WO_4_·2H_2_O (AR grade, Aladdin Co., Ltd., Shanghai, China), Na_2_SO_4_ (AR grade, Aladdin Co., Ltd., Shanghai, China), K_2_SO_4_ (AR grade, Aladdin Co., Ltd., Shanghai, China), terpineol (C_10_H_18_O) (AR grade, Fuchen Reagent Factory, Tianjin, China), hydrochloric acid (HCl; AR grade, Aladdin Co., Ltd., Shanghai, China), N,N-dimethylformamide (≥99.9%, CAS No.: 68-12-2, Macklin Biochemical Co., Ltd., Shanghai, China), and ethanol (≥99.8%, CAS No.: 64-17-5, Macklin Biochemical Co., Ltd., Shanghai, China) were employed in the current study. Additionally, graphene nanoplatelets were purchased from Suzhou Tanfeng Technology Co., Ltd. (Suzhou, China).

WO_3_ nanorods were prepared using a hydrothermal approach. For this purpose, Na_2_WO_4_·2H_2_O (2.08 g), Na_2_SO_4_ (0.25 g), and K_2_SO_4_ (0.3 g) were added to deionized water (40 mL). After stirring for 30 min, the solution pH was adjusted to 1.6 using 2 M conc. HCl. The precursor mixture was then transferred to a 100 mL Teflon-lined autoclave and subjected to a hydrothermal reaction at 180 °C for 24 h. After this time, the autoclave was allowed to cool naturally to room temperature (24 °C), and the obtained product was collected by centrifugation prior to washing alternately with deionized water and absolute ethanol six times. Finally, pure WO_3_ powder was obtained after drying at 60 °C for 8 h.

PPy particles were synthesized via a chemical oxidative polymerization method using pyrrole as the monomer and ammonium persulfate (APS) as the oxidant. Specifically, pyrrole (0.7 mL) and APS powder (2.28 g) were separately dissolved in 1 M dilute HCl (100 mL), and the APS solution was slowly added dropwise to the pyrrole solution in a beaker at a rate of 3 drops/s. The resulting mixture was stirred continuously for 3 h to yield the polymer precipitate. This product was subjected to vacuum filtration and washed repeatedly with absolute ethanol and deionized water six times. Finally, pure PPy powder was obtained after drying in a vacuum oven at 60 °C for 12 h.

**Figure 1:**
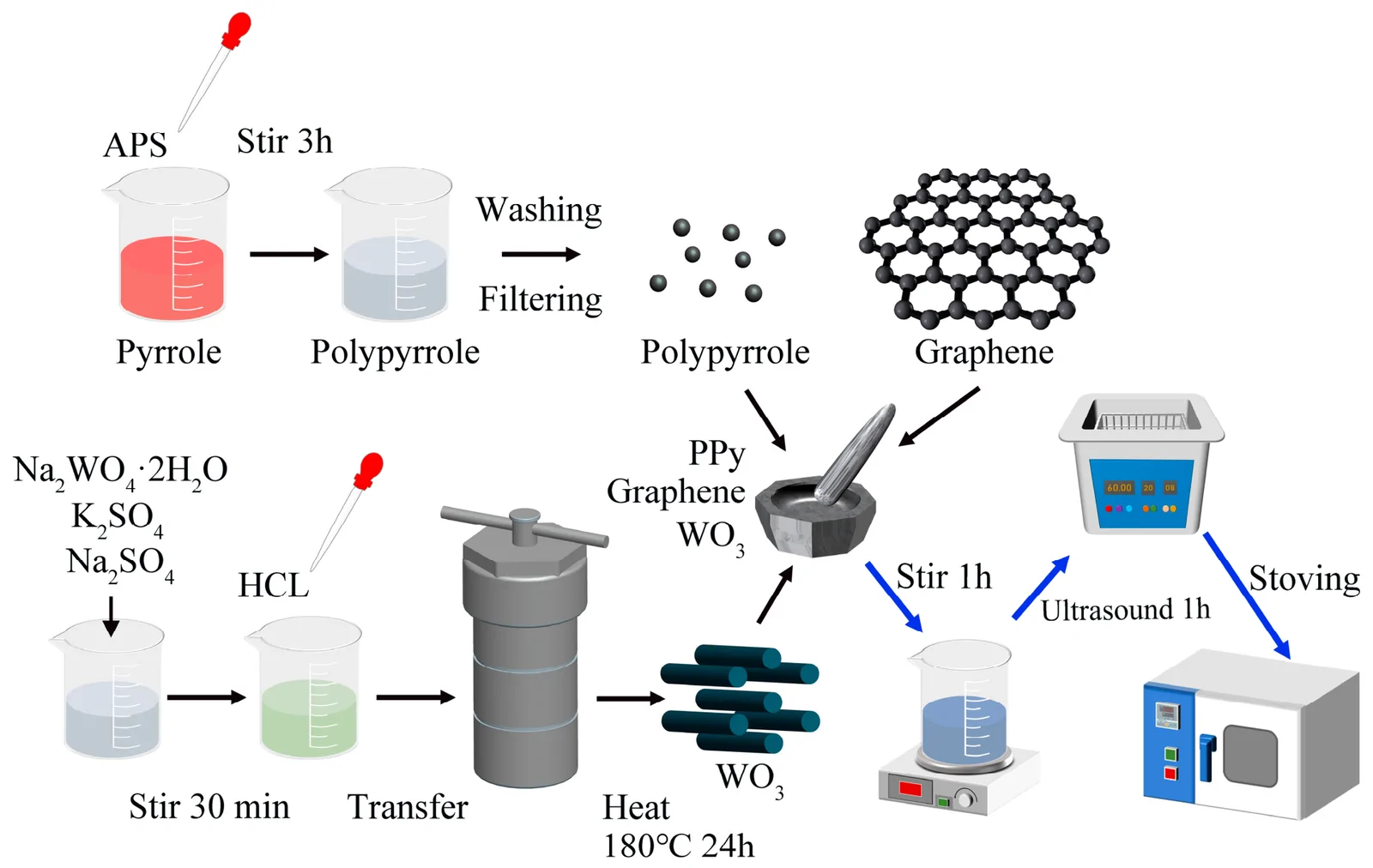
Preparation route of the PPy/Graphene/WO_3_ ternary composite.

The PPy/Graphene/WO_3_ ternary composite sensing material was prepared via a combination of physical mixing and ultrasonic dispersion. Specifically, the previously dried PPy powder, WO_3_ nanorods, and commercial graphene nanoplatelets were mixed according to a predetermined ratio and ground in a mortar. The mixed powder was then transferred to a beaker, to which an appropriate amount of deionized water (or solvent) was added. The mixture was magnetically stirred for 1 h and then subjected to ultrasonic treatment for 1 h to ensure complete interweaving and dispersion of the 1D WO_3_, 2D graphene, and PPy matrix. After treatment, the mixture was dried in an oven, ultimately yielding the ternary composite material. The gas-sensing performance of the resulting composite material was assessed by placing the sample in a mortar followed by the dropwise addition of terpineol (2 mL). The resulting mixture was ground in a mortar for 1 h to form a uniform paste, which was then coated onto an interdigitated electrode for subsequent testing. The preparation process is shown in Figure 1.

### 2.2. Material Characterization

**Figure 2:**
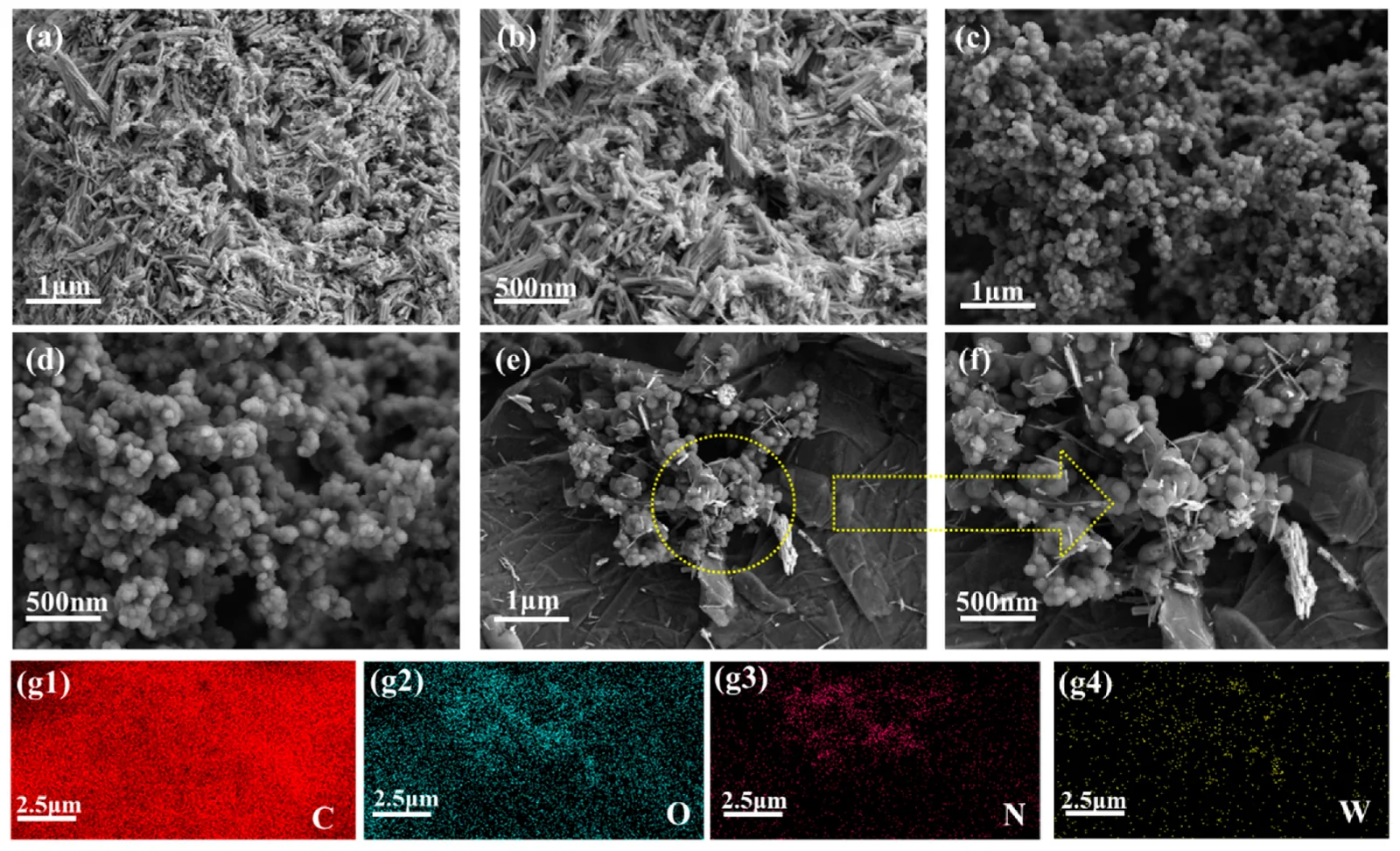
Microscopic morphology and elemental distribution of the pure WO_3_, pure PPy, and PPy/Graphene/WO_3_ ternary composite. (**a**,**b**) SEM images of the pure WO_3_ nanorods at different magnifications; (**c**,**d**) SEM images of the pure PPy nanospheres; (**e**,**f**) SEM images of the PPy/Graphene/WO_3_ ternary composite, showing the 3D network structure formed by the interwoven 0D PPy, 1D WO_3_, and 2D graphene components; (**g1**–**g4**) EDS elemental mapping images of C, O, N, and W for the composite.

Figure 2a,b illustrates the typical morphologies of the as-prepared tungsten oxide (WO_3_) nanorods at 1 μm and 500 nm scales, revealing numerous uniform nanostructures. Specifically, the WO_3_ nanorods interweave with one another to form a loose, porous network structure with a high specific surface area. Additionally, Figure 2c,d shows the corresponding SEM images of the as-prepared pure PPy. PPy exhibits a relatively regular spherical particle morphology with a degree of nanosphere agglomeration, which is commonly observed during PPy synthesis.

Figure 2e,f shows the morphologies of the composite material, which is composed of PPy doped with 1.5 wt% graphene and 6 wt% WO_3_, revealing significant morphological changes compared with the regular spherical morphology of pure PPy. Additionally, these images confirm that the 0D PPy, 1D WO_3_ nanorods, and 2D graphene nanoplatelets adhere to and interweave with one another, forming a more complex 3D conductive network structure corresponding to the ternary composite.

Raman spectroscopy was additionally used to examine the graphitic bands of PPy/Graphene and PPy/Graphene/WO_3_. TEM, HRTEM, SAED, and elemental mapping were used to identify nanosheet morphology, lattice fringes, interfacial contact, and the spatial distributions of C, N, O, and W.

**Figure 3:**
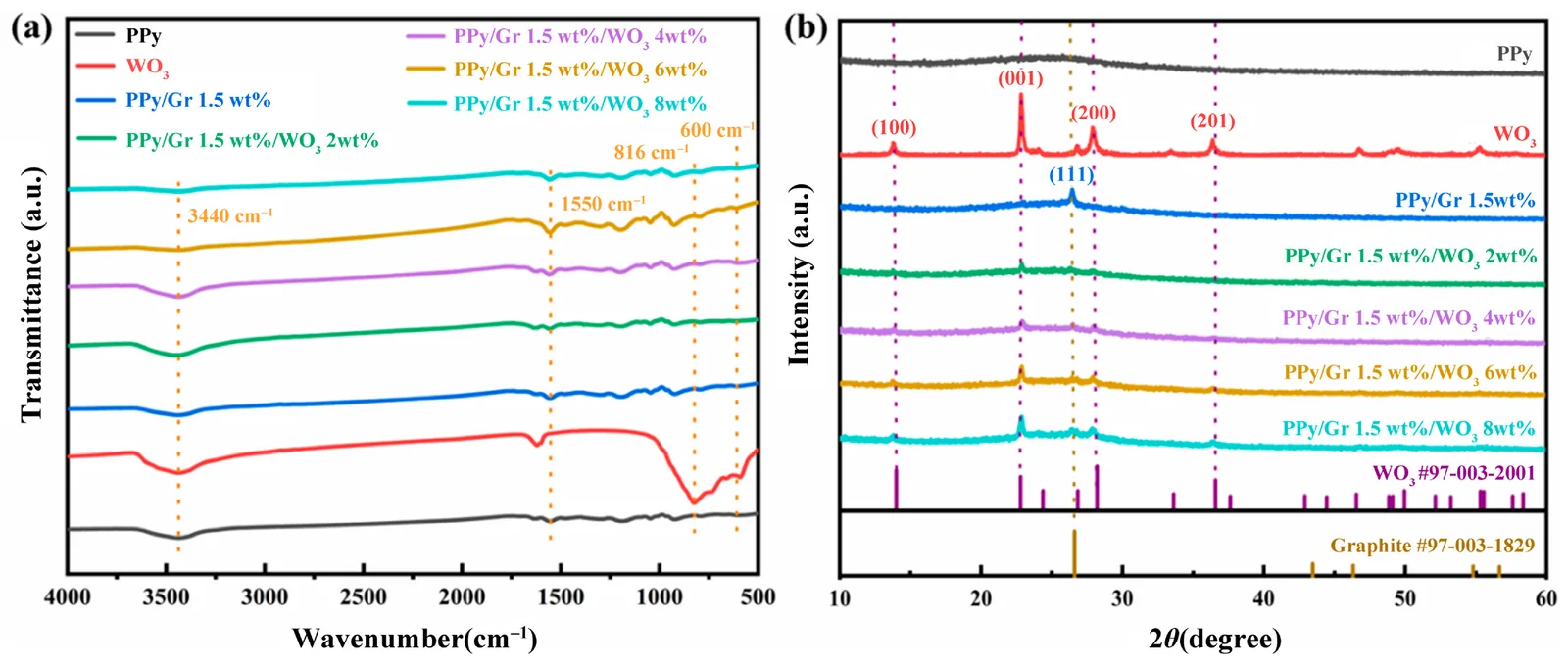
Structural characterization of PPy, WO_3_, and PPy/Graphene/WO_3_ composites. (**a**) FTIR spectra; (**b**) XRD patterns with reference reflections for hexagonal WO_3_ and rhombohedral graphite.

The morphologies of PPy, PPy/Graphene, and PPy/Graphene/WO_3_ were examined by field-emission scanning electron microscopy (FE-SEM; ZEISS Gemini SEM 300, Berlin, Germany) at an accelerating voltage of 3–15 kV. Their phase structures were analyzed by X-ray diffraction (XRD; Rigaku SmartLab 3 kW, Tokyo, Japan) using Cu Kα radiation over 2θ = 10–60°, consistent with the range displayed in Figure 3b. Functional groups were analyzed by Fourier-transform infrared (FTIR) spectroscopy (PerkinElmer 100, Los Angeles, CA, USA) over 400–4000 cm^−1^.

To examine the elemental distribution in the ternary composite, EDS elemental mapping was performed over the region shown in Figure 2f, with the corresponding maps presented in Figure 2(g1–g4). Carbon (Figure 2(g1)) and nitrogen (Figure 2(g3)), the characteristic elements of PPy, exhibit strongly overlapping distributions that outline the PPy matrix. Oxygen (Figure 2(g2)) and tungsten (Figure 2(g4)) originate primarily from the WO_3_ nanorods and are distributed throughout the composite network. The overlapping elemental distributions confirm the successful integration of graphene and WO_3_ nanorods within the PPy matrix.

Figure 3a presents the FTIR spectrum of the composite material. A broad peak can be observed at 3440 cm^−1^, attributed to the stretching vibration of the N–H bonds on the PPy chains and the O–H bonds of residual water molecules. The characteristic peak of PPy at 1550 cm^−1^ is associated with the stretching vibration of the PPy C=C bonds. The FTIR spectrum of WO_3_ is relatively simple, primarily exhibiting vibrations originating from metal–oxygen bonds in the low-wavenumber region. In particular, strong absorption peaks attributed to the W–O–W stretching vibration (~800 cm^−1^) and the W=O stretching vibration (~600 cm^−1^) can be clearly observed. These findings reveal that the composite material simultaneously possesses the organic functional groups of PPy and the inorganic metal oxide sites of WO_3_. This heterogeneous surface may therefore contribute to the highly sensitive detection of gases.

The XRD scan covered 2θ = 10–60°. Pure PPy exhibits a broad diffuse halo at 22–28°, whereas the principal peak of PPy/Graphene 1.5 wt% occurs at 26.61°, corresponding to an interplanar spacing of approximately 0.335 nm. According to Graphite PDF #97–003–1829, this peak is indexed as (111) in the rhombohedral setting and corresponds to the basal (003) interlayer reflection in the equivalent hexagonal setting, consistent with the approximately 0.34 nm graphitic-carbon interlayer spacing observed by HRTEM. The principal WO_3_ peaks at 13.98, 22.84, 28.14, and 36.52° are indexed as the (100), (001), (200), and (201) planes of hexagonal WO_3_, respectively, in agreement with PDF #97–003–2001. With increasing WO_3_ content, these reflections become more pronounced while the PPy diffuse halo and graphitic contribution remain visible, with no obvious additional crystalline impurity phases.

**Figure 4:**
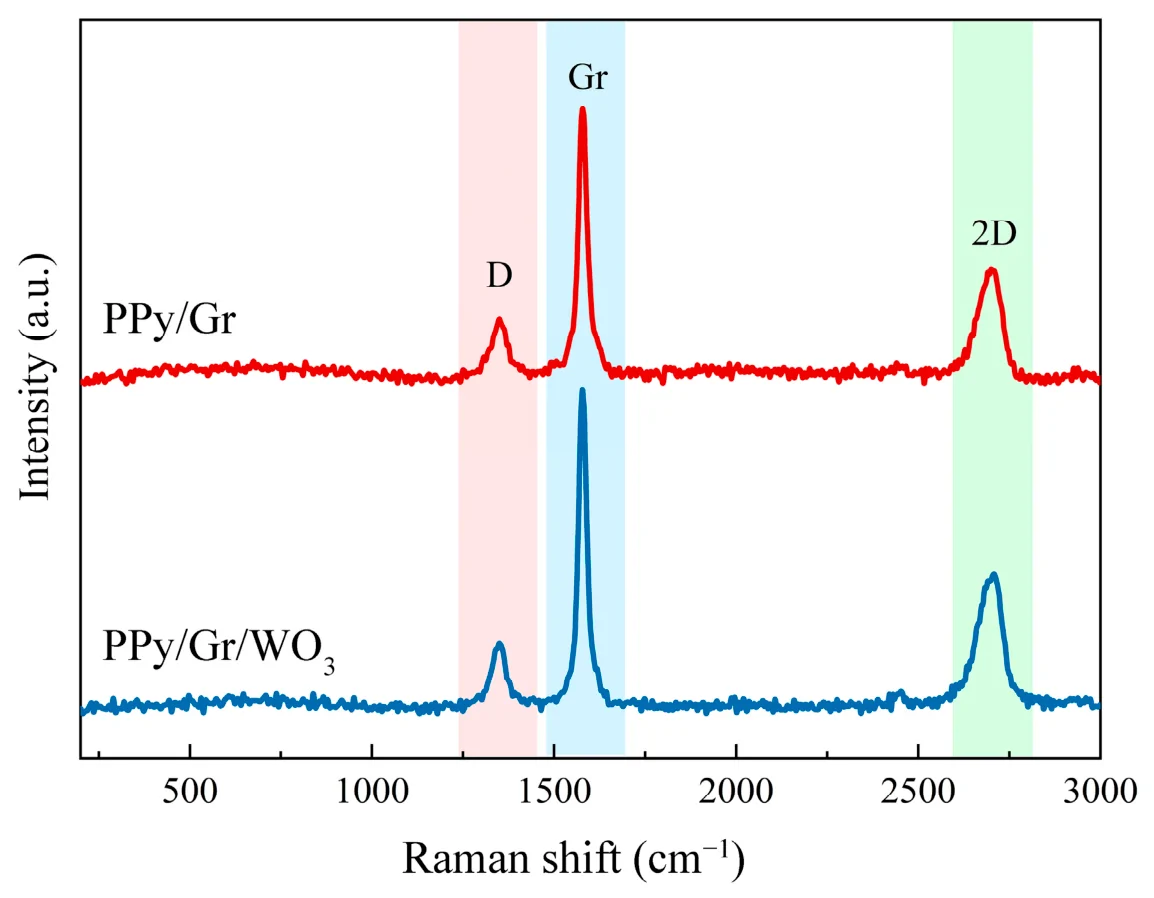
Raman spectra of PPy/Graphene and PPy/Graphene/WO_3_, showing the characteristic D, Gr, and 2D bands.

The Raman spectra in Figure 4 provide direct spectroscopic evidence for the graphene component. Both PPy/Graphene and PPy/Graphene/WO_3_ exhibit the characteristic D, G, and 2D bands. The D band is located at 1349.9 cm^−1^, the G band at 1577.6–1578.6 cm^−1^, and the 2D band at 2700.0–2708.1 cm^−1^, while the baseline-corrected I_D/I_G ratios range from 0.2005 to 0.2022. These results indicate that the graphitic sp^2^ framework is retained after composite preparation, and that the D band reflects defect and edge sites favorable for PPy and WO_3_ anchoring.

**Figure 5:**
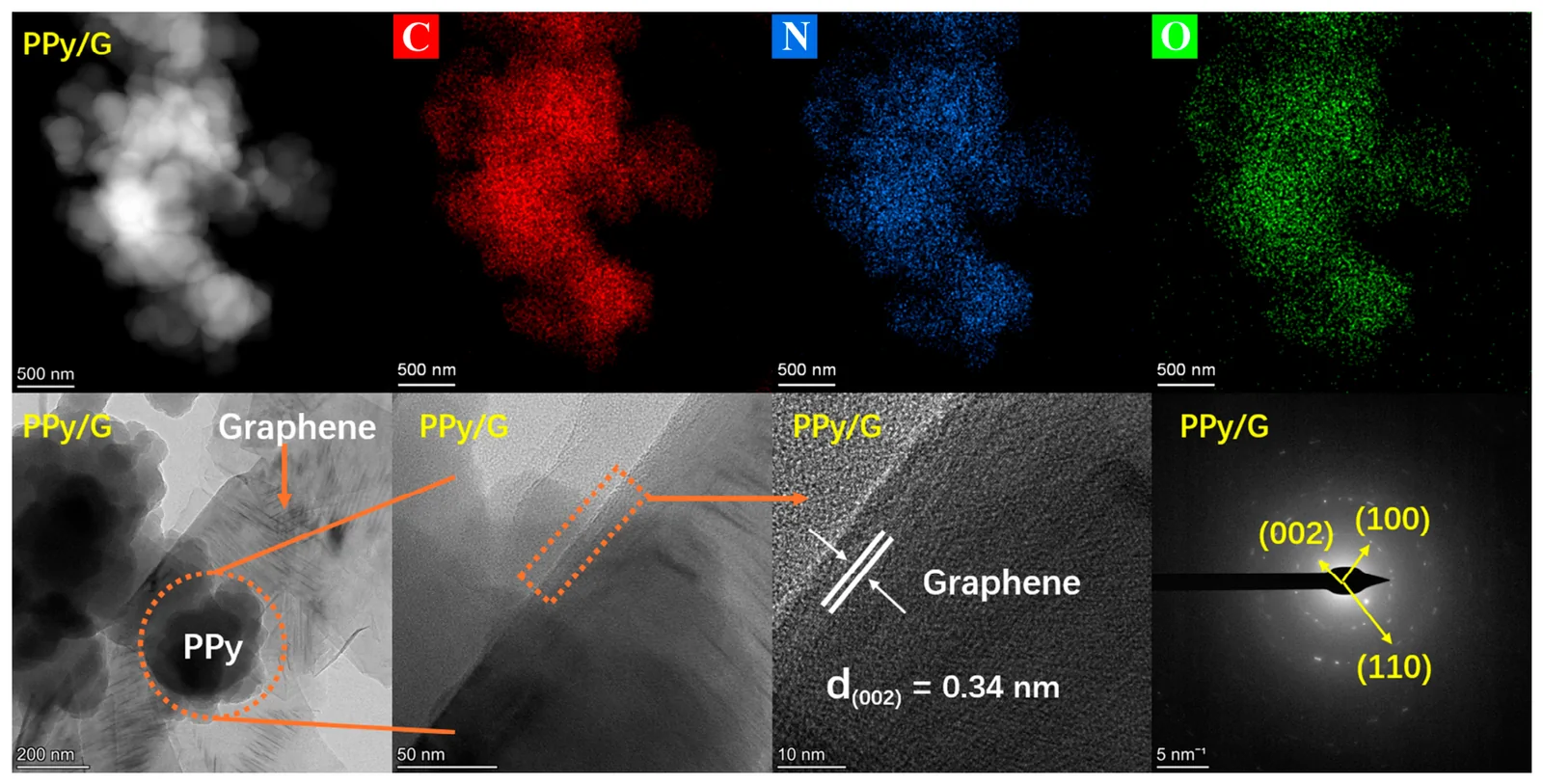
TEM/HRTEM, SAED, and elemental mapping of PPy/Graphene. The 0.34 nm lattice fringe is assigned to graphitic carbon (002).

TEM images of PPy/Graphene (Figure 5) show PPy particles distributed on thin, wrinkled carbon nanosheets. The overlapping C and N elemental maps indicate a close spatial integration of PPy with the graphitic sheets. HRTEM reveals a lattice spacing of 0.34 nm, assigned to the graphitic-carbon (002) plane, while the corresponding SAED pattern contains the (002), (100), and (110) reflections.

**Figure 6:**
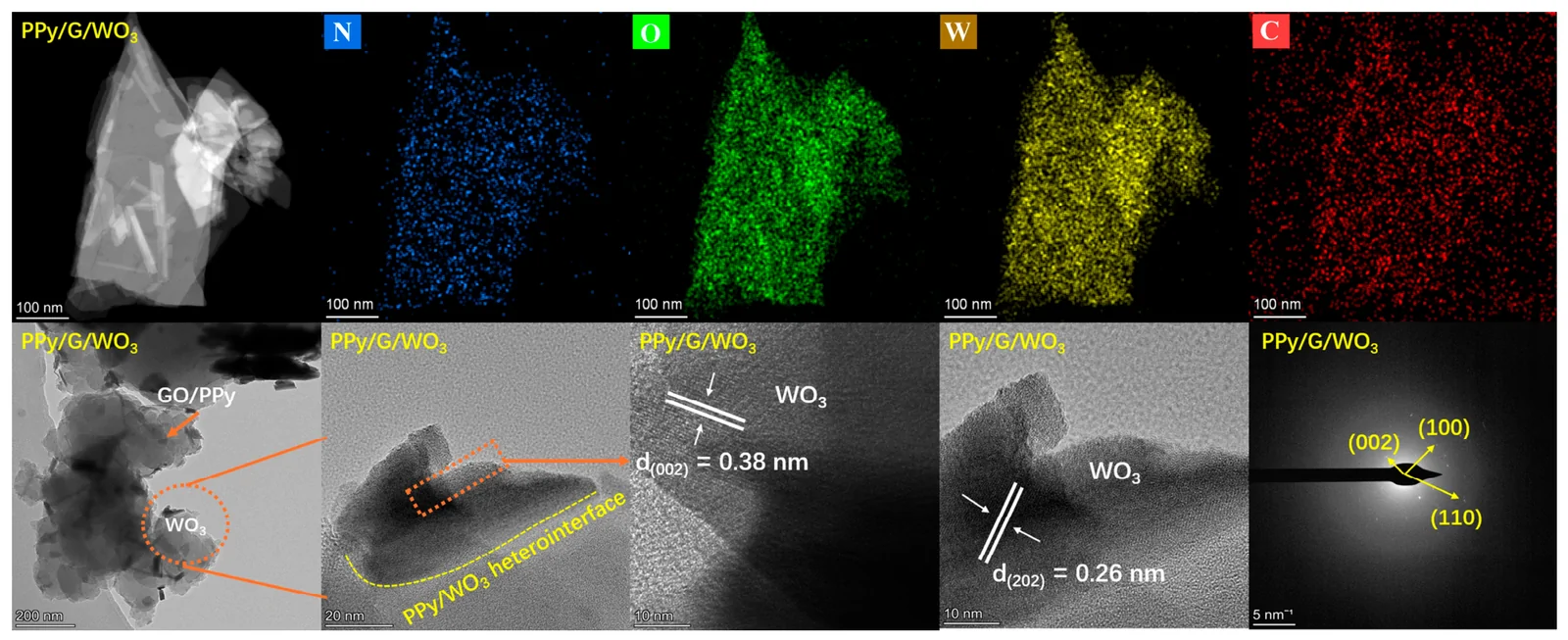
TEM/HRTEM, SAED, and C/N/O/W elemental mapping of the PPy/Graphene/WO_3_ composite. The 0.38 and 0.26 nm lattice fringes are assigned to the WO_3_ (002) and (202) planes, respectively.

In the ternary composite (Figure 6), crystalline WO_3_ domains are in direct contact with the less-crystalline PPy/Graphene matrix. Lattice spacings of 0.38 and 0.26 nm are assigned to the WO_3_ (002) and (202) planes, respectively. The WO_3_ lattice fringes terminate at a continuous PPy/Graphene boundary without an obvious interfacial gap, and W/O-rich regions are spatially adjacent to the C/N-containing matrix. These observations confirm an intimate nanoscale heterointerface.

### 2.3. Gas-Sensing Performance

**Figure 7:**
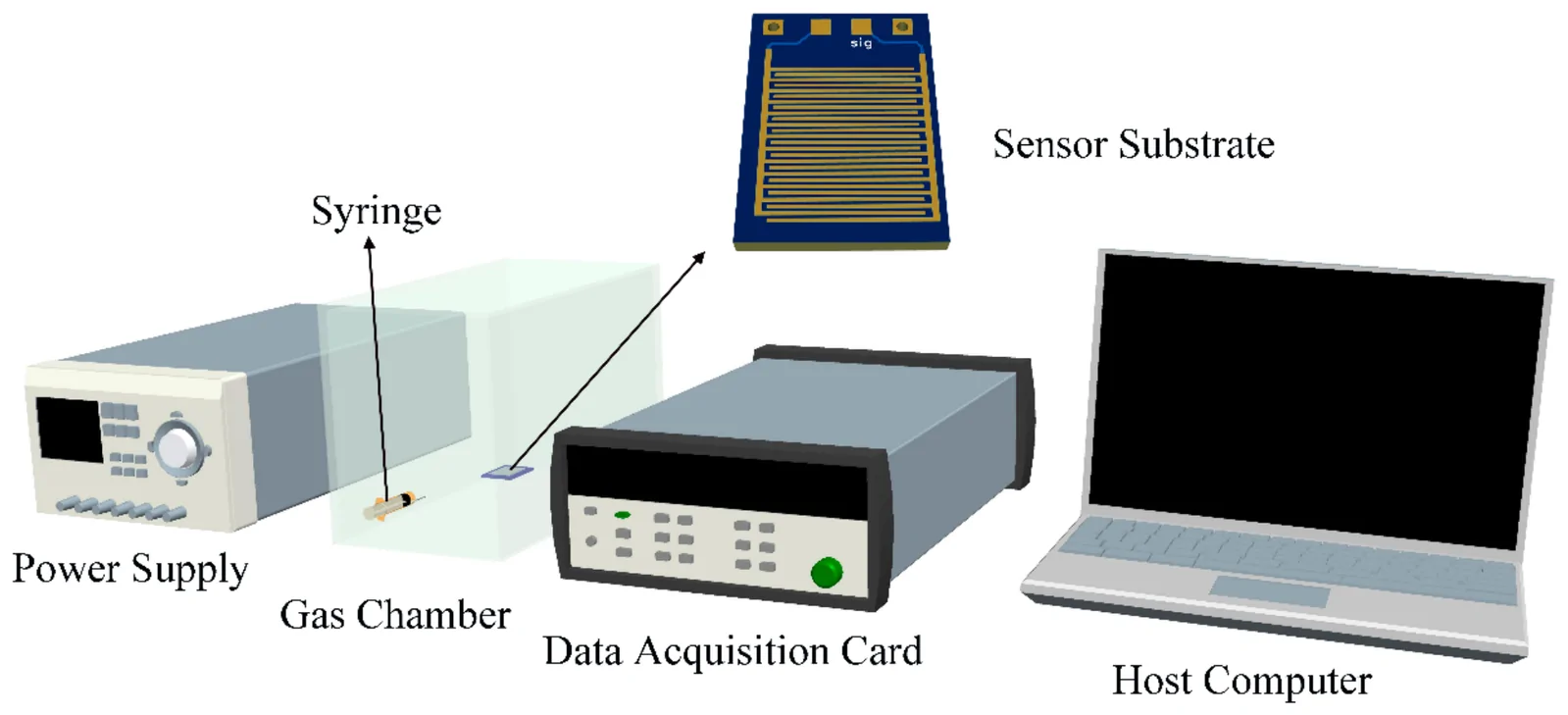
Schematic of the gas-sensing test system.

The gas-sensing performance of the devices was evaluated within a standard static gas-sensing test system. As illustrated in Figure 7, the test system primarily consists of four core components, namely, a regulated power supply, a sealed testing gas chamber, a data acquisition card, and a host computer terminal. During the testing process, the interdigitated electrode sensor substrate, which was coated with the composite sensing material, was fixed inside the sealed gas chamber, and a stable working bias voltage was provided using an external power supply. During dynamic testing, a microsyringe was utilized to inject a precisely measured volume of the target gas or volatile test liquid into the chamber, achieving precise control over the target gas concentration within the test chamber. Upon adsorption or redox reactions between the target gas molecules and the sensing material on the device surface, the resulting minute variations in macroscopic electrical parameters (such as resistance or current) were captured in real time using a high-precision data acquisition card. These signals were synchronously transmitted to the host computer software for visual monitoring, data storage, and analysis, enabling the dynamic gas-sensing response characteristics to be monitored. The sensor response was quantified using the relative resistance change as *S* (%) = *ΔR*/*R*_a_ × 100% = |*R*_g_ − *R*_a_|/*R*_a_ × 100%, where *R*_a_ denotes the stable resistance in air immediately before target-gas exposure and *R*_g_ denotes the resistance measured after the response reaches a quasi-steady state under the target gas.

**Figure 8:**
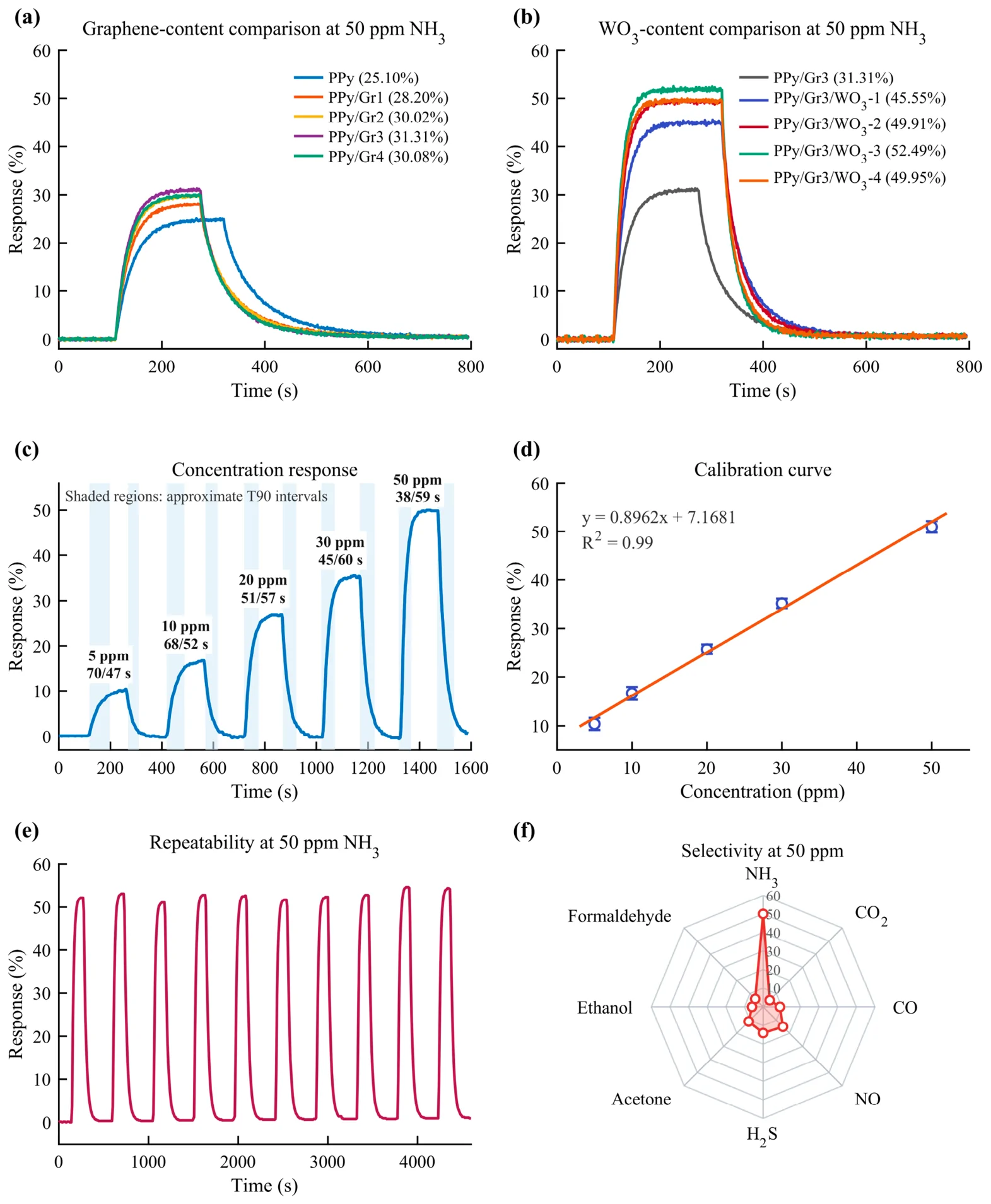
Gas-sensing performance of the PPy/Graphene/WO_3_ sensors. (**a**) Responses of binary PPy/Graphene sensors to 50 ppm NH_3_; (**b**) responses of ternary sensors with different WO_3_ contents to 50 ppm NH_3_; (**c**) dynamic responses to 5–50 ppm NH_3_; (**d**) response-concentration calibration; (**e**) repeatability over 10 cycles at 50 ppm NH_3_; and (**f**) selectivity toward NH_3_.

To investigate the effect of graphene content on gas sensor performance, five binary sensors with different graphene mass fractions (0–2%)—denoted as PPy (0 wt%), PPy/Gr1 (0.5 wt%), PPy/Gr2 (1 wt%), PPy/Gr3 (1.5 wt%), and PPy/Gr4 (2 wt%)—were tested in the presence of 50 ppm NH_3_ at room temperature. The response and recovery curves shown in Figure 8a reveal that the PPy sensor exhibited a response of 25.10%. Initially, the response increased with increasing graphene content, giving a maximum response value of 31.31% for the PPy/Gr3 (1.5 wt%) sensor. This indicates that the introduction of graphene significantly improved sensor performance; however, increasing the graphene doping amount to 2 wt% had a detrimental effect on sensor performance. The optimal composition for the binary sensor was therefore determined to be PPy/Gr3 (1.5 wt%). To achieve a further performance enhancement, four ternary sensors with different WO_3_ mass fractions (2–8%)—denoted as PPy/Gr3/WO_3_-1 (2 wt%), PPy/Gr3/WO_3_-2 (4 wt%), PPy/Gr3/WO_3_-3 (6 wt%), and PPy/Gr3/WO_3_-4 (8 wt%)—were tested in the presence of 50 ppm NH_3_ at room temperature. The response and recovery curves shown in Figure 8b reveal that the sensing performance was enhanced upon increasing the WO_3_ doping ratio, giving a maximum response value of 52.49% for the PPy/Gr3/WO_3_-3 sensor (i.e., 1.68 times that of PPy/Gr3).

Using the T_90_ criterion, the measured response/recovery times at room temperature were 70/47, 68/52, 51/57, 45/60, and 38/59 s for 5, 10, 20, 30, and 50 ppm NH_3_, respectively. The response time decreased from 70 to 38 s with increasing concentration, whereas the recovery time remained within 47–60 s. At 50 ppm, the present sensor showed shorter response and recovery times than a room-temperature WO_3_/PPy sensor (50/70 s) [17]. Related studies have examined ZnO-based acetone sensing [28], WS_2_-based acetone sensing [29], and RGO/SnO_2_ H_2_/LPG sensing [30]. Because their target analytes and operating conditions differ, they are treated here as contextual benchmarks rather than direct NH_3_ comparisons. A graphene/PPy composite has also been reported for rapid room-temperature NH_3_ sensing [31]. In this context, the present device combines room-temperature operation with directly measured T_90_ response/recovery dynamics over 5–50 ppm NH_3_ and a 52.49% response at 50 ppm.

To comprehensively evaluate the core performance of the ternary sensor, repeatability experiments were performed. Specifically, the sensor was continuously exposed to a 50 ppm NH_3_ environment over 10 cycles at room temperature. As shown in Figure 8e, the response curve for each cycle exhibited similar peak values and recovery trajectories, indicating that the sensor possesses good repeatability. Additionally, the sensor selectivity was evaluated upon exposure to various potential interfering gases. As shown in Figure 8f, despite the presence of multiple gases, the sensor produced a strong response to NH_3_ (approximately 50%), whereas the responses to CO_2_, CO, NO, H_2_S, acetone, ethanol, and formaldehyde were approximately 5%, 9%, 15%, 14%, 11%, 6%, and 6%, respectively. Thus, the NH_3_ response was at least 3.3 times that of the largest interfering-gas response under the tested 50 ppm condition. The corresponding gas-specific dynamic response traces and response/recovery characteristics are provided in Supplementary Figure S4.

**Figure 9:**
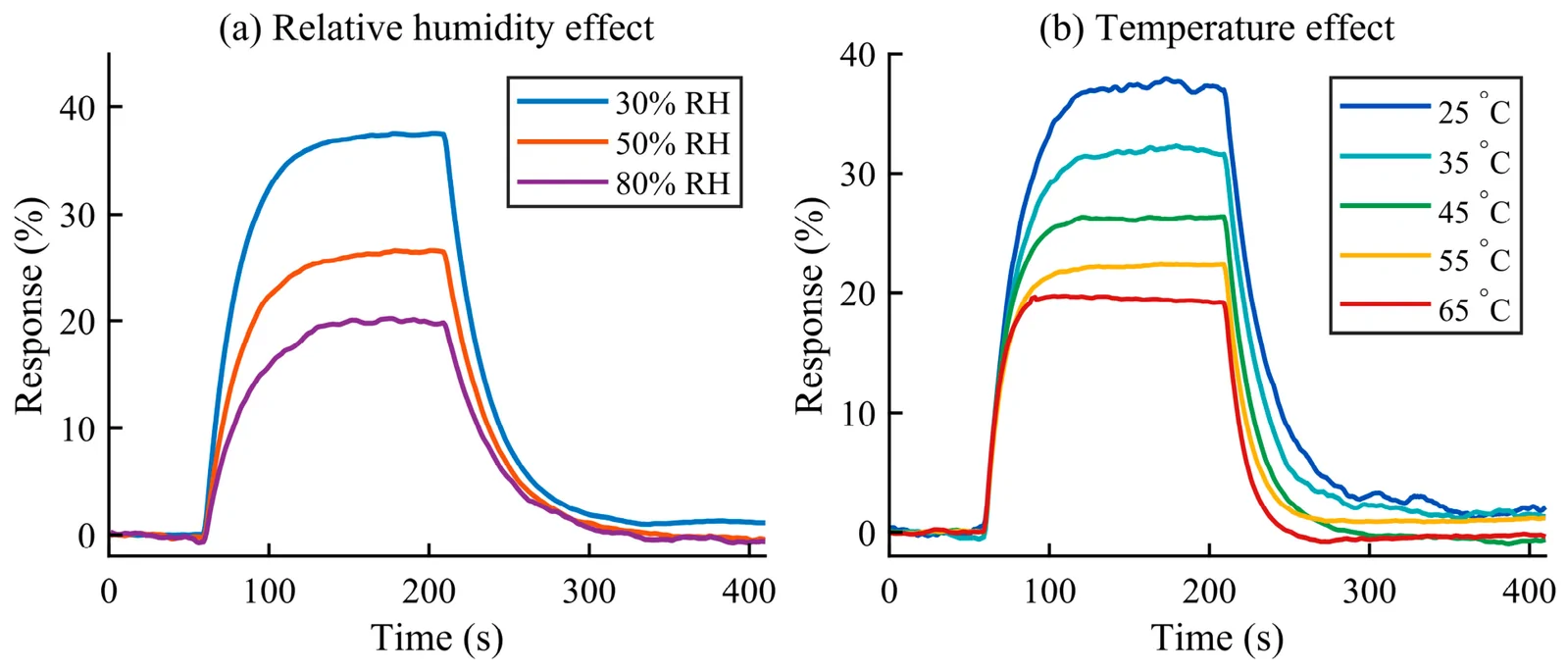
Effects of environmental conditions on the response of the PPy/Graphene/WO_3_ sensor to 30 ppm NH_3_. (**a**) Relative humidity and (**b**) operating temperature.

The environmental dependence of the optimal PPy/Graphene/WO_3_ sensor was experimentally evaluated at a fixed NH_3_ concentration of 30 ppm. Each trace was corrected using the mean pre-exposure baseline over 1–40 s, and the response/recovery times were extracted using the T_90_ criterion. As shown in Figure 9, increasing the relative humidity from 30% to 80% RH decreased the steady-state response from 37.396% to 19.986%, while the response/recovery times increased from 46/64 to 59/72 s. This trend is attributed to humidity-induced changes in the carbon-based conductive network and the competitive adsorption of H_2_O and NH_3_ at active sites [32,33]. Increasing the temperature from 25 to 65 °C decreased the steady-state response from 37.371% to 19.385% and the apparent sensitivity at 30 ppm from 1.246 to 0.646% ppm^−1^, while shortening the response/recovery times from 41/73 to 19/25 s. Elevated temperature therefore accelerates interfacial kinetics and desorption but reduces effective adsorption and sensitivity; consequently, 25 °C, which provided the highest sensitivity among the tested temperatures, was selected as the operating temperature.

**Figure 10:**
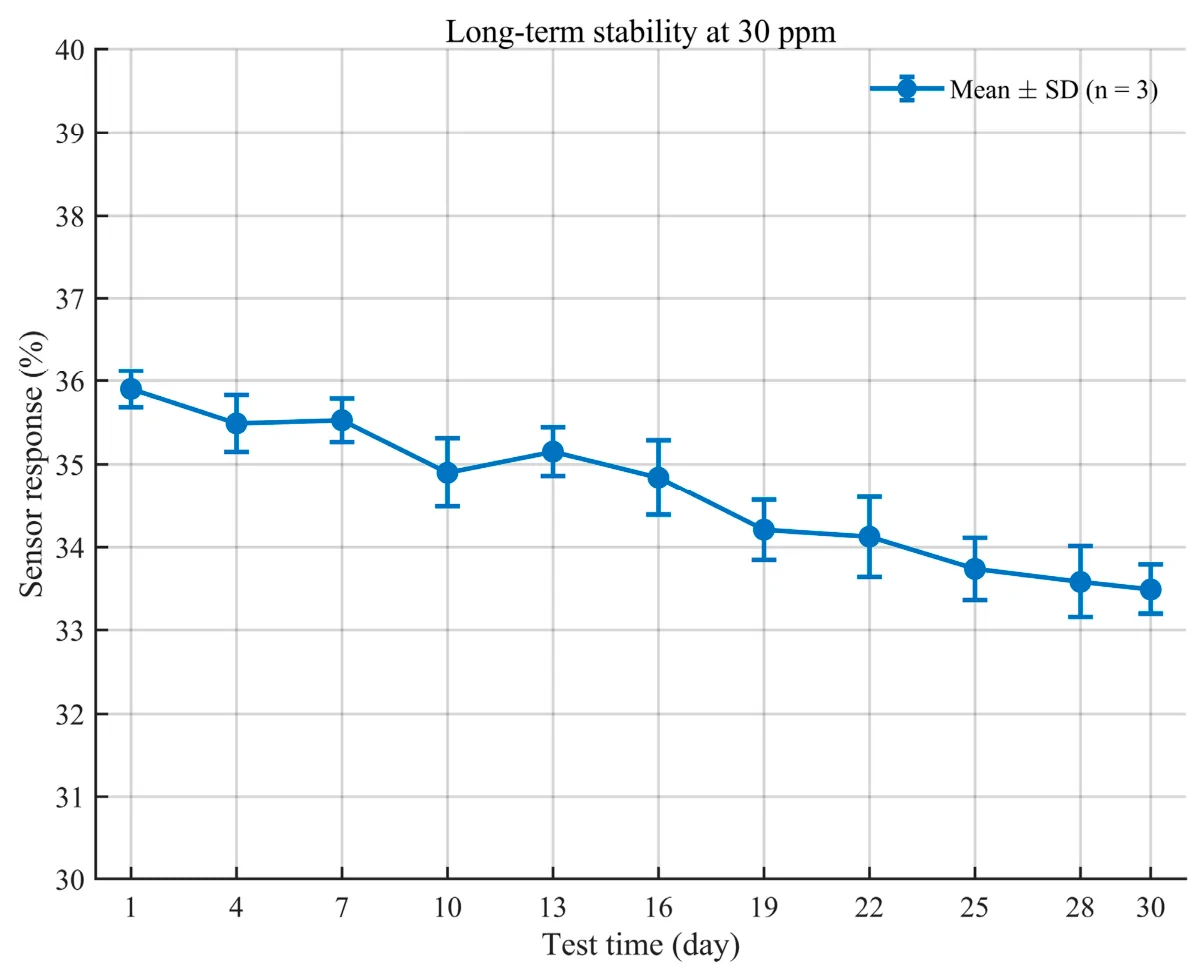
Thirty-day stability of the PPy/Graphene/WO_3_ sensor at 30 ppm NH_3_. Error bars represent the standard deviation of three parallel tests.

Long-term stability was evaluated at room temperature under 30 ppm NH_3_ for 30 days. Measurements were performed every 3 days, with three parallel tests at each time point; the error bars denote the standard deviation. As shown in Figure 10, the response gradually decreased from 35.90 ± 0.24% on day 1 to 33.50 ± 0.32% on day 30, corresponding to an absolute decrease of approximately 2.40 percentage points, a relative attenuation of 6.70%, and a 30-day response retention of 93.30%. The standard deviations at all time points were approximately 0.24–0.50 percentage points, with no abrupt response loss or marked increase in dispersion.

The calculated limit of detection was retained at 0.0470 ppm and determined as LOD = 3 σ_blank/S, where σ_blank = 0.0140% is the standard deviation of the stable air-baseline response and S = 0.8962% ppm^−1^ is the slope of the 5–50 ppm calibration curve. Thus, LOD = 3 × 0.0140%/(0.8962% ppm^−1^) = 0.0469 ppm, reported as 0.0470 ppm. Additional dynamic exposure tests were conducted at 0.1, 0.5, 1, and 5 ppm NH_3_, producing peak responses of approximately 1.57%, 2.87%, 6.14%, and 11.69%, respectively (Supplementary Figure S1). The response at 0.1 ppm clearly exceeded the baseline fluctuations and returned close to the baseline after NH_3_ removal, directly validating detection at 0.1 ppm and supporting sub-ppm sensing. Accordingly, 0.0470 ppm is the calculated LOD, while 0.1 ppm is the lowest concentration directly validated by the supplementary experiments.

### 2.4. Sensing Mechanism

**Figure 11:**
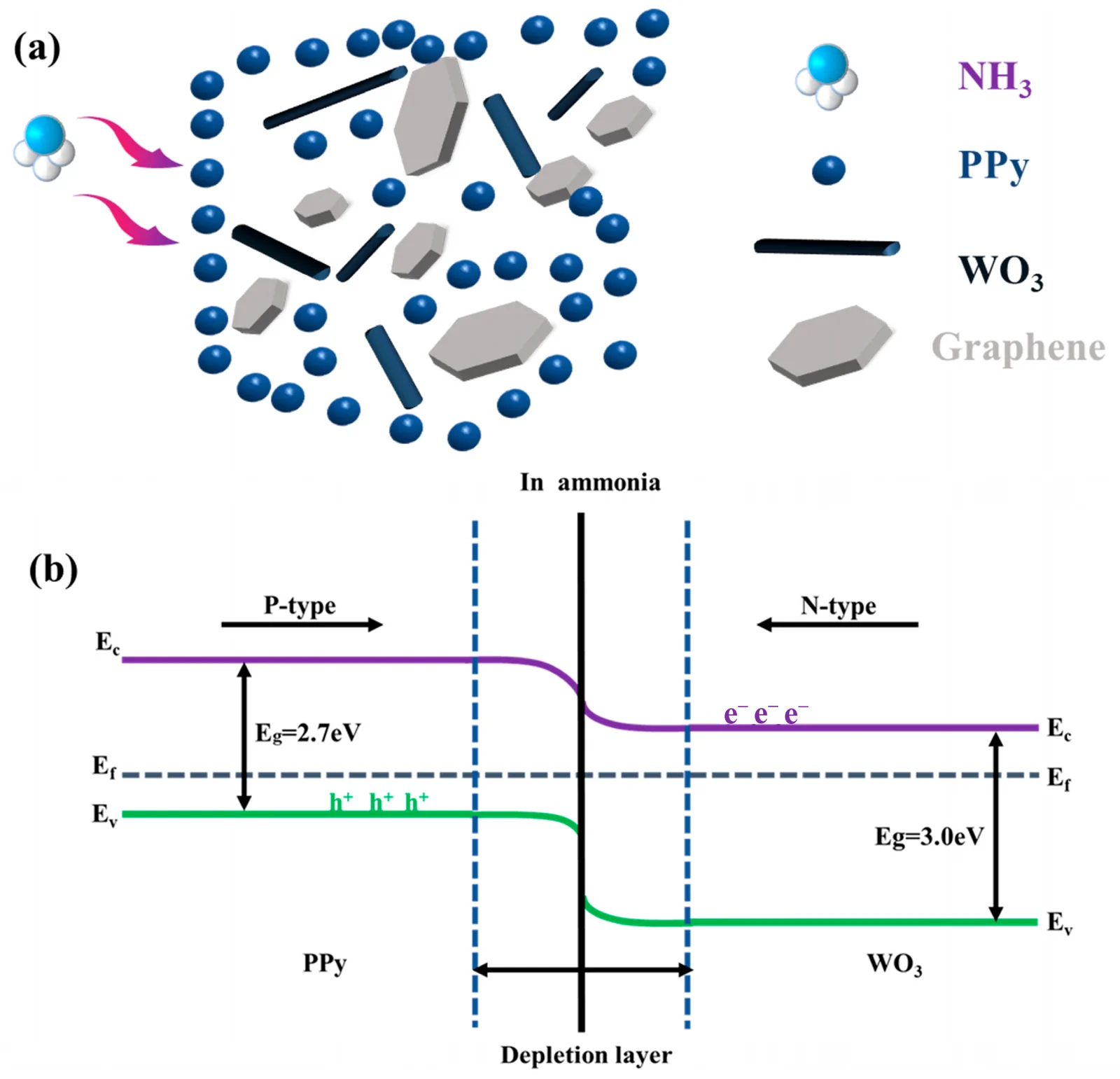
Gas-sensing mechanism of the PPy/Graphene/WO_3_ ternary composite. (**a**) Gas adsorption and charge transport in the 3D heterogeneous network; (**b**) interfacial band structure of the PPy/WO_3_ p-n heterojunction.

The room-temperature NH_3_-sensing performance of the PPy/Graphene/WO_3_ ternary composite is attributed to the combined structural and charge-transport roles of the 0D–1D–2D porous network. As shown in Figure 11, Raman D/G/2D bands support the retention of the graphitic sp^2^ framework, while the D band indicates defect and edge sites favorable for PPy and WO_3_ anchoring. TEM/HRTEM and elemental mapping show PPy on wrinkled graphene sheets and crystalline WO_3_ in direct contact with the PPy/Graphene matrix without an obvious interfacial gap. These features provide accessible adsorption sites and continuous pathways for propagating local conductance perturbations. Graphene serves mainly as the conductive backbone, PPy supplies the dominant room-temperature chemiresistive response, and WO_3_ provides oxide-related adsorption sites.

The intimate boundary observed by HRTEM provides the structural prerequisite for carrier redistribution at the interface between p-type PPy and n-type WO_3_ [34,35], consistent with the mechanism reported for a related rGO-WO_3_-PPy system [36]. In air, oxygen adsorbs at WO_3_-related sites and forms anionic oxygen species, predominantly O_2_^−^ at room temperature, as expressed in Equation (1). The resulting surface charge contributes to the baseline resistance and interfacial barrier:

O_2_ (gas) + e^−^ → O_2_^−^ (ads) (1)

Upon NH_3_ exposure, NH_3_ reacts with pre-adsorbed oxygen and releases electrons while also interacting with the oxidized PPy backbone to induce de-doping, as represented in Equations (2) and (3):

4NH_3_ (gas) + 3O_2_^−^ (ads) → 2N_2_ (gas) + 6H_2_O (gas) + 3e^−^ (2)

PPy^+^ + NH_3_ → PPy^0^ + NH_3_^+^ (3)

The resulting electron supply decreases the majority-hole concentration in PPy and increases the resistance. The accompanying charge redistribution modulates the PPy/WO_3_ interfacial barrier, while the graphene network transmits the local conductance change through the composite.

## 3. 3D Detection System and PINN Architecture

**Figure 12:**
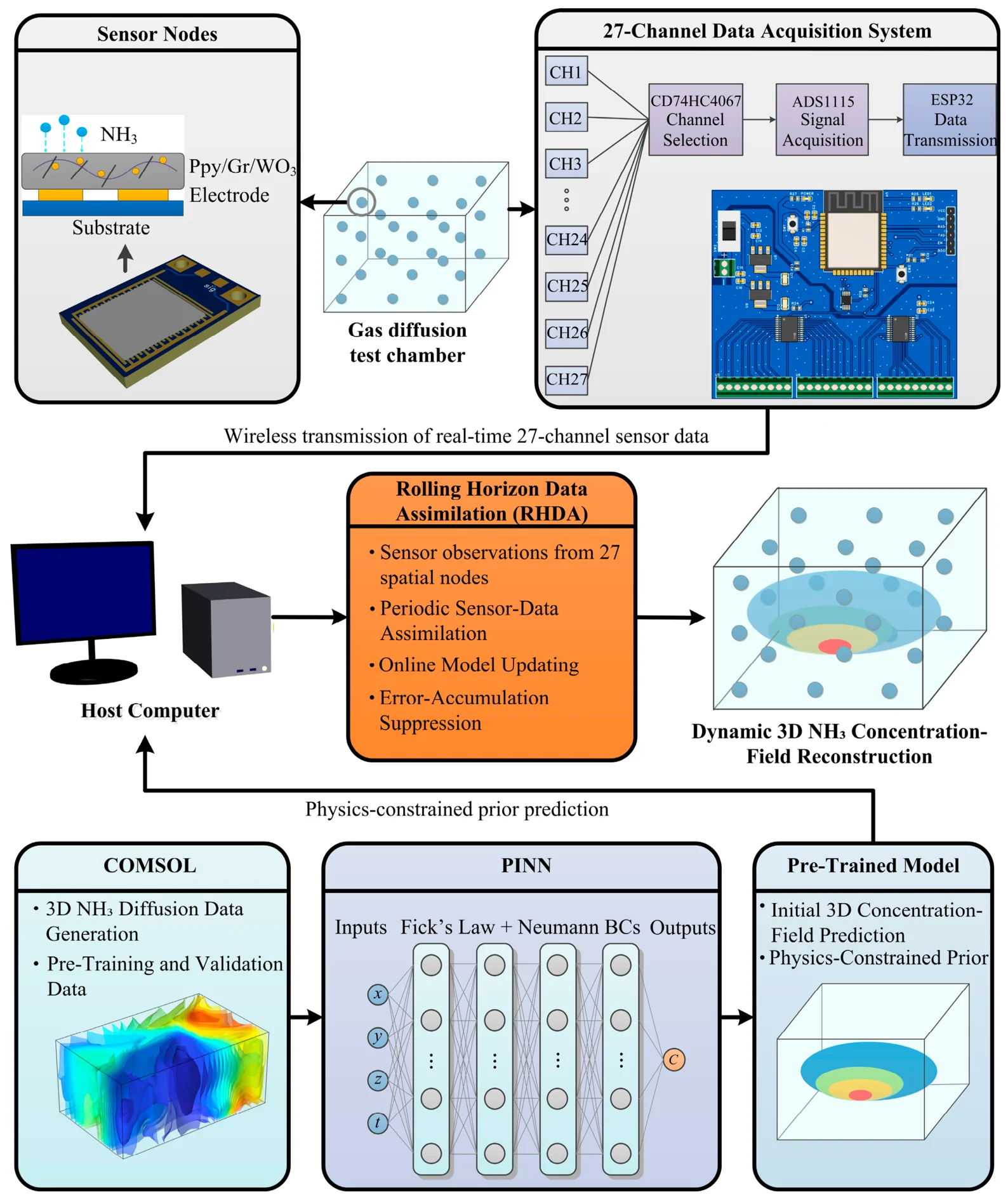
Integrated material-sensing, multichannel-acquisition, PINN, and RHDA framework for dynamic three-dimensional NH_3_ concentration-field reconstruction.

Figure 12 summarizes the integrated material sensing–multichannel acquisition–physics-based modeling–rolling horizon data assimilation–three-dimensional reconstruction framework. The framework does not merely place the sensing material and reconstruction algorithm in parallel; instead, RHDA serves as the coupling interface between sensor observations and the physical model. PPy/Graphene/WO_3_ nodes provide local NH_3_ measurements with spatial coordinates and time stamps, and the 27-channel hardware enables synchronous acquisition and transmission. The PINN provides a full-field prior constrained by the governing diffusion equation, which RHDA periodically corrects using the node observations. In this way, discrete sensor measurements are continuously assimilated into the physical model to dynamically reconstruct a continuous three-dimensional NH_3_ concentration field.

### 3.1. 27-Channel Data Acquisition System

**Figure 13:**
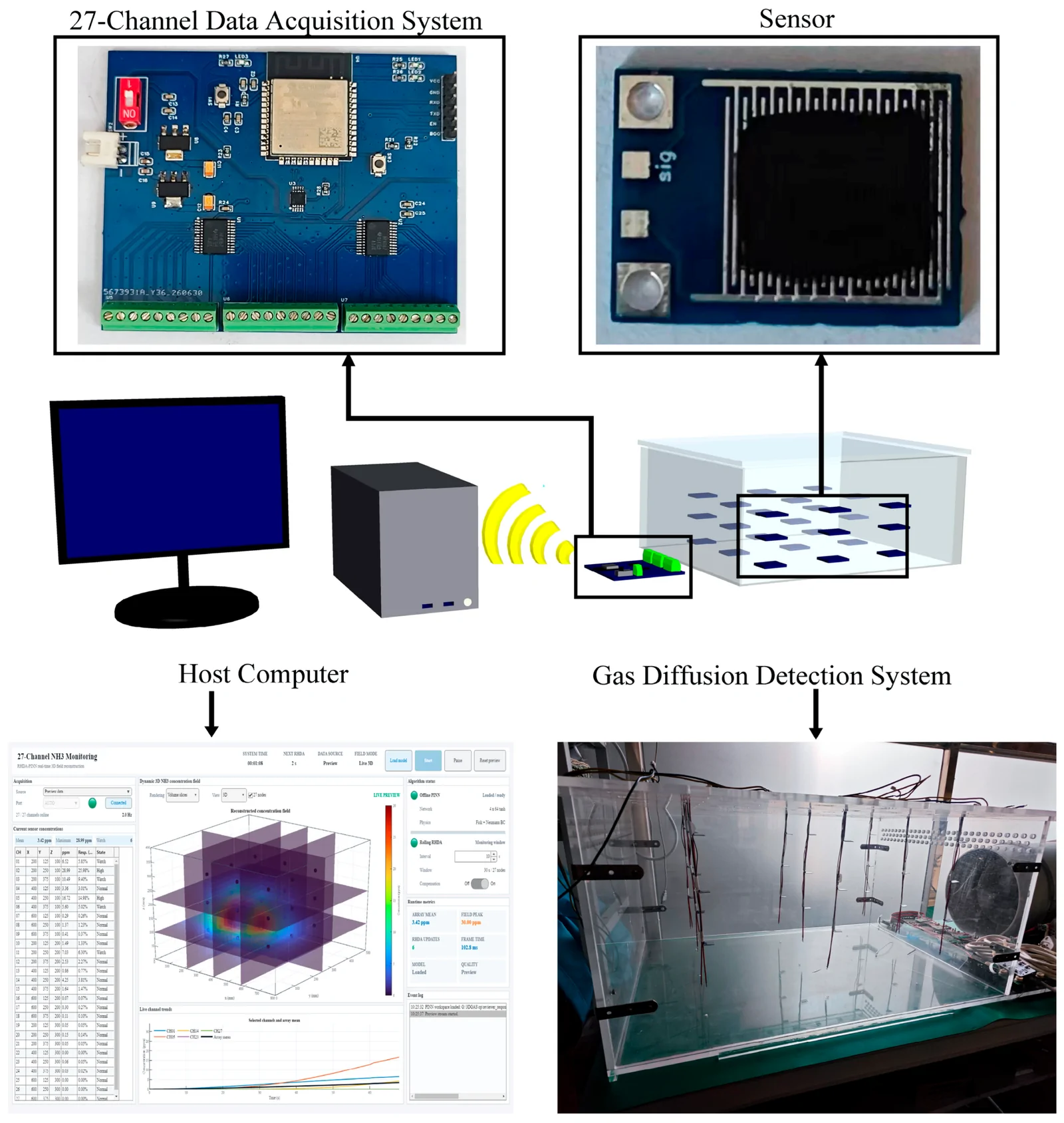
Three-dimensional gas-diffusion detection system and 27-channel data-acquisition platform. The upper panels show the 27-channel data-acquisition board and the PPy/Graphene/WO3 sensor; the central schematic shows the host-computer-linked acquisition and sensing architecture; and the lower panels show the monitoring interface and experimental chamber.

To verify the practicability of the PPy/Graphene/WO_3_ ternary nanocomposite, a centralized data acquisition system based on time-division multiplexing was constructed to synchronously monitor multiple gas nodes within a confined space. As shown in Figure 13, this provides hardware support for capturing the highly nonlinear 3D spatiotemporal evolution of NH_3_ leakage. The hardware architecture employs an ESP32 microcontroller (Espressif Systems (Shanghai) Co., Ltd., Shanghai, China) as the main control core, interfacing with a 27-channel PPy/Graphene/WO_3_ sensor array at the front end via cascaded analog multiplexers (CD74HC4067SM96, Texas Instruments Incorporated, Dallas, TX, USA). To address the minute resistance variations induced by low-concentration NH_3_, a 16-bit high-precision analog-to-digital converter (ADS1115IDGSR, Texas Instruments Incorporated, Dallas, TX, USA) and a programmable gain amplifier were introduced into the system to enhance signal resolution. During test operations, the main control module sequentially polls each sensor channel with a fixed period. It then converts the acquired analog signals into a time-stamped global state matrix, which is transmitted to the host computer via Bluetooth. With respect to the data synchronization mechanism, the system achieves high-speed, seamless switching and data acquisition across the 27 sensor channels by utilizing the internal RDY (conversion ready) hardware interrupt pin of the ADS1115 to trigger the external interrupt service routine of the ESP32. This guarantees strict temporal alignment between the physical sensing data and the time-domain PINN model. This system effectively mitigates the timing skew of asynchronous multi-node sampling, thereby providing synchronized, high-SNR physical inputs for the online data assimilation of the back-end PINN model.

### 3.2. Physics-Informed Neural Network Design

Benefiting from the low limit of detection and high sensitivity of the sensor array, the 27 discrete hardware sensing nodes can serve as highly reliable “data anchors,” capturing early, minute leak-induced concentration gradients within the space with a high SNR [23,24]. Driven by this real-time sensing data stream, a PINN-based gas-diffusion inference model was proposed. This model embeds Fick’s law of diffusion into network training, achieving accurate reconstruction of the 3D global flow field from localized discrete sensing data [26].

**Figure 14:**
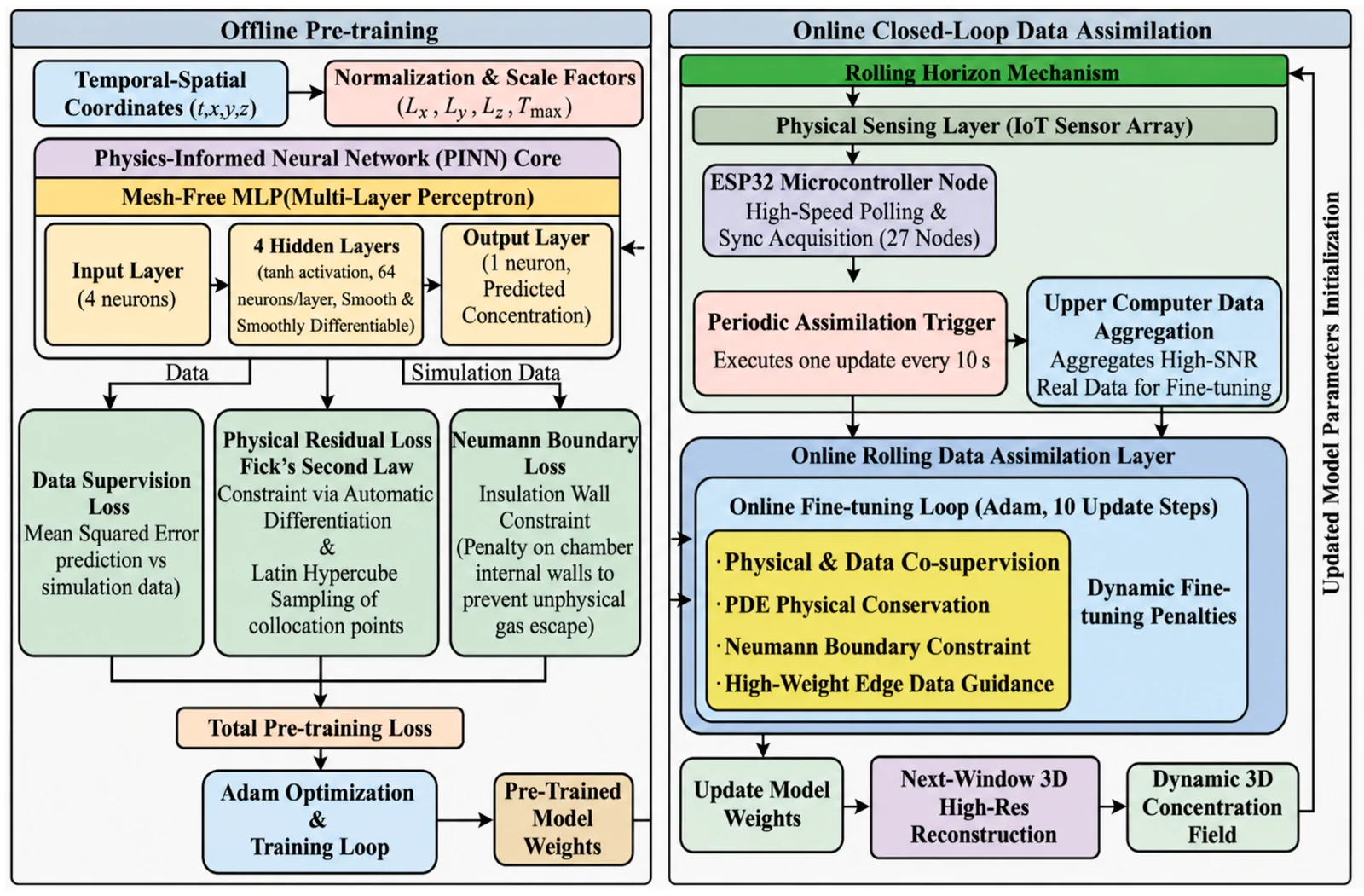
Closed-loop PINN workflow combining physical constraints, offline pre-training, and rolling horizon data assimilation.

As shown in Figure 14, to balance computational efficiency and accuracy in real-time inference, two core modules were incorporated into the overall architecture of the diffusion model: (1) offline construction and pre-training of the PINN, and (2) online closed-loop data assimilation based on a rolling horizon mechanism. Initially, the system utilizes CFD simulation data as prior knowledge for the offline pre-training of the diffusion model. Subsequently, combined with the rolling horizon mechanism, the model is fine-tuned to achieve 3D, high-resolution dynamic concentration field reconstruction for the subsequent time window. The COMSOL Multiphysics 6.2 configuration, simulated spatiotemporal evolution, and reproducible dataset-construction protocol used for offline PINN pre-training are provided in Section S5 and Supplementary Figure S5.

The material and reconstruction sections are connected through the proposed sensing–inference workflow. The experimentally measured sensor response, dynamic characteristics, environmental dependence, and stability define the requirements of the observation layer, while PINN/RHDA evaluates how sparse node concentrations constrain a continuous concentration field.

### 3.3. Physical Modeling and Governing Equations

This study focuses on the interior of a sealed confined space under constant temperature and humidity conditions, where the indoor background air velocity is extremely low and the NH_3_ leak is released slowly, introducing no significant external momentum or thermodynamic disturbances. For computationally efficient online reconstruction, the transport process is represented in the PINN by a reduced-order, diffusion-dominated constraint based on Fick’s second law [37,38]. In the PINN framework, this baseline physical process is governed by a reduced-order Fick’s second law constraint. Its spatiotemporal evolution in a 3D Cartesian coordinate system can be described by the following partial differential equation:

(4) $$\frac{𝜕c}{𝜕t}=D\left(\frac{{𝜕}^{2}c}{𝜕x^{2}}+\frac{{𝜕}^{2}c}{𝜕y^{2}}+\frac{{𝜕}^{2}c}{𝜕z^{2}}\right)$$

where *c*(*t*, *x*, *y*, *z*) is the gas concentration at time *t* and spatial coordinates (*x*, *y*, *z*), and *D* is the diffusion coefficient of the gas in air. The reduced-order constraint is mainly applicable to sealed or weak-flow environments and to stages in which diffusion is the dominant transport mechanism. To accurately predict the long-term accumulation of gas within the confined chamber using the diffusion model and prevent the non-physical phenomenon of “escaping through walls” in the predicted field, Neumann boundary conditions must be imposed on the six physical inner walls of the cavity [39,40]. Specifically, the concentration gradient of the gas at the boundaries along the wall-normal vector (**n**) is zero [41]:

(5) $$\frac{𝜕c}{𝜕n}=0,\;(x,y,z)\in \Gamma_{wall}$$

where $\Gamma_{\mathrm{wall}}$ denotes the set of physical boundaries of the chamber.

Purely data-driven deep learning models are highly prone to overfitting with sparse monitoring data [42]. To achieve super-resolution inversion of the continuous gas diffusion field, this study constructs a PINN surrogate model, establishing a nonlinear approximation function $\hat{c}(t,x,y,z;\theta )$ from spatiotemporal coordinates to the target concentration. This function is essentially a composite mapping parameterized by a multi-layer fully connected feedforward network, whose mathematical form can be expressed as:

(6) $$\hat{c}(t,x,y,z;\theta )=N(t,x,y,z;\theta ).$$

The internal structure of the network consists of a series of nested linear transformations and nonlinear activation functions. The input vector comprises the normalized spatiotemporal coordinates $x={\left[t,x,y,z\right]}^{T}$. The network performs feature extraction through *L* layers of nonlinear transformations, where the output h*_k_* of each layer *k* follows the iterative relationship: $h_{k}=\sigma \left(W_{k}h_{k-1}+b_{k}\right)$. In this expression, **W***_k_* and **b***_k_* represent the trainable weight matrix and bias vector, respectively, which together constitute the network parameter *θ*. To satisfy the computational requirements for the second-order partial derivatives involved in the partial differential equation (PDE) of Equation (4), this model employs the highly smooth and infinitely differentiable tanh activation function across all hidden layers. This architecture guarantees the continuity and second-order differentiability of the function throughout the entire spatiotemporal domain. Consequently, it enables the system to utilize automatic differentiation techniques to precisely internalize Fick’s law and the boundary conditions as regularization penalty terms within the network.

### 3.4. Multi-Dimensional Composite Loss Function Design

The core of the PINN lies in its composite loss function $L_{\mathrm{total}}$. By rationally allocating weight factors $\lambda_{\mathrm{data}}$, $\lambda_{\mathrm{pde}}$, and $\lambda_{\mathrm{bc}}$, the model achieves high-precision mapping of the 3D spatiotemporal diffusion flow field during the offline pre-training stage [43]. In this study, $L_{\mathrm{total}}$ is defined as the weighted sum of the data loss, physical residual loss, and boundary loss:

(7) $$L_{\mathrm{total}}=\lambda_{\mathrm{data}}L_{\mathrm{data}}+\lambda_{\mathrm{pde}}L_{\mathrm{pde}}+\lambda_{\mathrm{bc}}L_{\mathrm{bc}}.$$

With respect to the data supervision loss ($L_{\mathrm{data}}$), this term is used to quantify the MSE between the predicted concentration of the PINN and the reference concentration *c*_true_. The source of *c*_true_ exhibits two-stage evolutionary characteristics. Specifically, during the offline pre-training stage, *c*_true_ is derived from finite element simulation results, aiming to inject macroscopic prior knowledge of the physical flow field into the model. However, upon entering the rolling horizon online data assimilation phase, *c*_true_ represents the node observations used during rolling horizon assimilation. In the controlled reconstruction experiments reported in this study, these observations were sampled from the COMSOL reference field at the 27 predefined node locations. In the intended online deployment, the same loss term accepts calibrated observations from the physical 27-channel sensing system. This design incorporates discrete node data through a common RHDA interface to correct theoretical prediction deviations. Its discrete mathematical expression is as follows:

(8) $$L_{\mathrm{data}\;}=\frac{1}{N_{\mathrm{data}\;}}\sum_{i=1}^{N_{\mathrm{data}\;}}\;{\left|\hat{c}\left(t_{i},x_{i}\right)-c_{\mathrm{true}\;}\left(t_{i},x_{i}\right)\right|}^{2}$$

Regarding the physical residual loss ($L_{\mathrm{pde}}$), to ensure numerical stability and accelerate convergence during the backpropagation process of the neural network, the input features must be normalized to the range of [0, 1] [26]. Therefore, a time-scaling factor *T*_max_ and spatial scaling factors (*L*_x_, *L*_y_, and *L*_z_) must be introduced to restore the true physical gradients using the chain rule:

(9) $$L_{pde}=\frac{1}{N_{pde}}\sum_{i=1}^{N_{pde}}{\left|\frac{1}{T_{\max}}\frac{𝜕\hat{c}}{𝜕t}-D\left(\frac{1}{L_{x}^{2}}\frac{{𝜕}^{2}\hat{c}}{𝜕x^{2}}+\frac{1}{L_{y}^{2}}\frac{{𝜕}^{2}\hat{c}}{𝜕y^{2}}+\frac{1}{L_{z}^{2}}\frac{{𝜕}^{2}\hat{c}}{𝜕z^{2}}\right)\right|}^{2}$$

where $N_{\mathrm{pde}}$ denotes the number of internal physical collocation points randomly deployed in the spatiotemporal domain via LHS.

For the Neumann boundary loss ($L_{bc}$), in the airtight chamber of the confined space, gas molecules are unable to penetrate the rigid physical walls to undergo mass exchange. Thus, the Neumann boundary condition is incorporated into the total loss function to forcibly impose this zero-flux wall constraint in the PINN, enabling the model to autonomously learn and strictly adhere to the global law of mass conservation within the fluid domain [44,45]. The discrete mathematical expression for this term is as follows:

(10) $$L_{bc}=\frac{1}{N_{bc}}\sum_{j=1}^{6}\sum_{i=1}^{N_{bc,j}}{\left|\nabla \hat{c}\cdot n_{j}\right|}^{2}$$

where *N*_bc_ denotes the total number of boundary sampling points, i.e., the number of test points randomly extracted from all inner walls of the confined chamber for error calculation. Additionally, **n***_j_* represents the outward normal vector of the *j*-th wall, which is used to constrain the gas diffusion flux perpendicular to the wall to zero.

**Figure 15:**
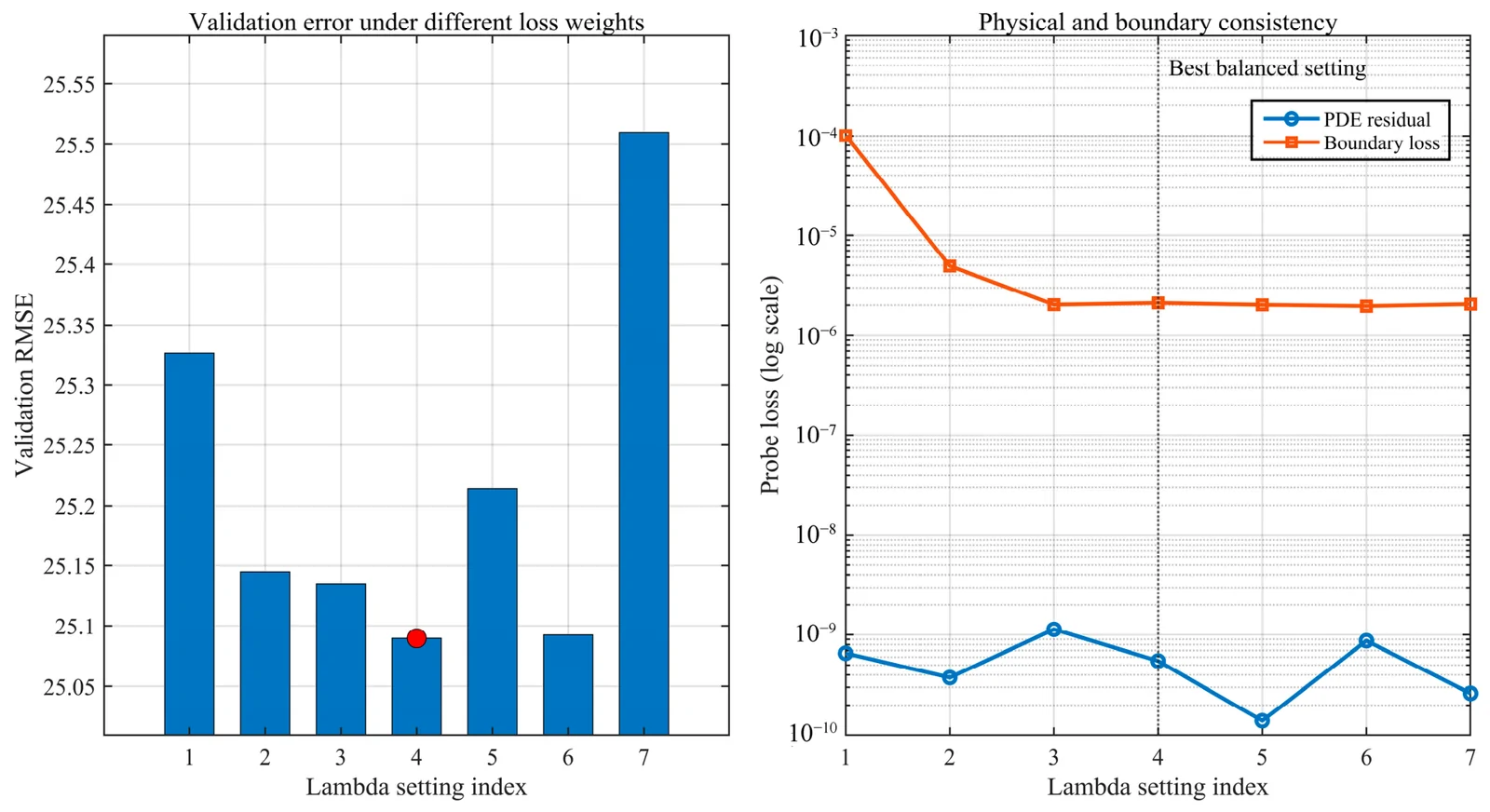
Sensitivity of validation RMSE, PDE residual, and boundary loss to the PINN loss weights. The dashed line marks the selected setting.

To evaluate the sensitivity of the PINN to the loss weights, the same four-layer, 64-neuron tanh network was tested using seven weight combinations while the data split, initialization, and mini-batches were held fixed. With λ_data fixed at 1, the validation RMSE varied only from 25.09 to 25.51, whereas the data-only setting produced a markedly larger boundary loss. The best balanced setting was λ_data = 1, λ_PDE = 1000, and λ_BC = 10, which yielded the lowest validation RMSE of 25.09 together with a PDE residual of 5.50 × 10^−10^ and a boundary loss of 2.11 × 10^−6^. An excessive boundary weight of λ_BC = 500 increased the validation error, indicating that overly strong boundary penalization reduces data fidelity. These results are summarized in Figure 15.

### 3.5. Rolling Horizon Data Assimilation Framework

Recent studies on IoT monitoring, PINN-based field reconstruction, and real-time gas-concentration prediction provide methodological context for the sensing–inference workflow [46,47,48]. To address the issues of error accumulation and temporal divergence inherent in static PINN models under purely offline-driven (open-loop) modes when confronting unknown source perturbations or environmental disturbances, the RHDA framework was developed. Upon the introduction of the RHDA mechanism, the PINN model system transitioned from its original open-loop inference mode to a closed-loop system equipped with real-time feedback and rectification capabilities. To control computational overhead while ensuring dynamic monitoring accuracy, the system configures the online assimilation process to execute every 10 s. For any discrete online assimilation timestamp *t_k_* (*t_k_* = 10, 20, 30, …s), the data supervision loss term $L_{\mathrm{data}}^{RHDA}$ is reconstructed as the mean squared error between the predicted values of the network and the transient node observations supplied through the RHDA interface. In the controlled reconstruction experiments reported here, these observations were sampled from the COMSOL reference field at the predefined node locations. Due to the inherent kinetic delay in the surface chemical adsorption/desorption process of PPy-based gas-sensing materials, their actual response time is typically longer than the RHDA assimilation period (*Δt* = 10 s). If the raw output of the sensors is directly employed for high-frequency assimilation, severe phase-lag errors can be introduced. To address this, prior to feeding the observational data into the PINN, this study introduces a first-order sensor dynamic model for time-delay compensation:

(11) $$c_{i}^{\mathrm{comp}}\left(t_{k}\right)=c_{i}^{\mathrm{obs}}\left(t_{k}\right)+\tau_{i}\frac{dc_{i}^{\mathrm{obs}}\left(t_{k}\right)}{dt}$$

where $c_{i}^{\mathrm{obs}}$ is the raw observed concentration of the *i*-th sensor, and *τ* is the sensor time constant. Using the measured T90 = 45 s at 30 ppm, τ = T90/ln(10) = 19.54 s according to the conventional response-time definition [49]; $\frac{dc_{i}^{cbs}}{dt}$ is the real-time rate of change in the response, and $c_{i}^{\mathrm{comp}}$ is the compensated transient true concentration estimate. Through this first-order feedforward compensation, the system reduces the phase-lag effect of material hysteresis on the concentration signal. The compensated value $c_{i}^{\mathrm{comp}}$ then replaces the raw readings to calculate the data supervision loss in RHDA:

(12) $$L_{\mathrm{data}}^{RHDA}\left(t_{k},\theta \right)=\frac{1}{N_{s}}\sum_{i=1}^{N_{s}}{\left|\hat{c}\left(t_{k},x_{i};\theta \right)-c_{i}^{\mathrm{comp}}\left(t_{k}\right)\right|}^{2}$$

where Ns denotes the total number of observation nodes used in each RHDA update; these are predefined virtual nodes in the controlled evaluation and correspond to physical sensing nodes in the intended deployment; xi represents the fixed 3D spatial coordinates of the i-th observation node; and is the transient concentration predicted by the PINN at time tk and spatial location xi. This closed-loop assimilation process adheres to the following principles:

(1) Few-step online update: Upon receiving new observations, the model performs 10 Adam update steps. On the tested RTX 3070 platform, one RHDA update required 1.158 s on average, which is substantially shorter than the 10 s assimilation interval.

(2) Preservation of physical laws: During the fine-tuning process, the physical residual and boundary losses remain activated in real time. Thus, while the network utilizes rolling node observations to rectify deviations, the output global flow field must rigorously comply with physical conservation laws, effectively avoiding the local overfitting caused by purely data-driven updates.

After each controlled update, the revised network reconstructs the continuous concentration field for the next time window. The reported synchronization and error reduction refer to agreement with the COMSOL reference field under virtual node observations. The online composite loss is:

(13) $$L_{\mathrm{total}}^{RHDA}\left(t_{k},\theta \right)=\lambda_{\mathrm{data}}^{RHDA}L_{\mathrm{data}}^{RHDA}\left(t_{k},\theta \right)+\lambda_{\mathrm{pde}}L_{\mathrm{pde}}+\lambda_{bc}L_{bc}\left(\lambda_{data}^{RHDA}\gg \lambda_{pde},\lambda_{bc}\right)$$

**Figure 16:**
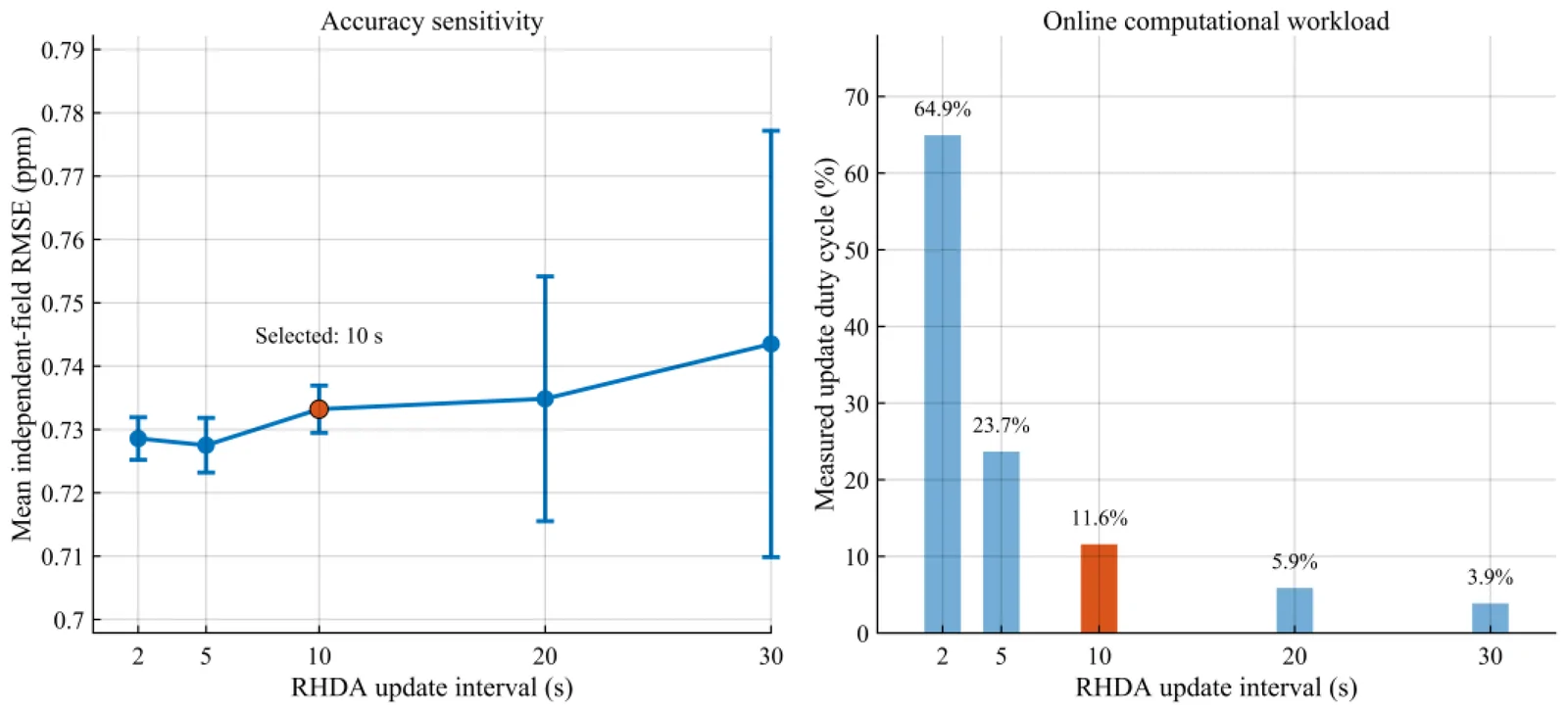
Effect of the RHDA update interval on independent-field RMSE and the measured duty cycle of a 10-step online update.

To compare the effects of different RHDA sampling intervals, intervals of 2, 5, 10, 20, and 30 s were evaluated using the same 4 × 64 PINN, the same independent COMSOL field points, and 10 Adam steps per update with the physical and boundary losses retained. The corresponding mean independent-field RMSE values were 0.7286 ± 0.0034, 0.7275 ± 0.0043, 0.7332 ± 0.0037, 0.7349 ± 0.0193, and 0.7435 ± 0.0337 ppm. Increasing the interval from 5 to 10 s raised the mean RMSE by only 0.78% while reducing the update duty cycle from 23.67% to 11.58%. The 2 s interval provided no stable accuracy gain but increased the duty cycle to 64.94%. At 20–30 s, the mean error increased modestly, but temporal variability increased and the worst-case RMSE at 30 s reached 0.8026 ppm. Therefore, 10 s was selected as a balance among dynamic stability, online latency, and edge workload rather than as the absolute minimum-RMSE setting, as shown in Figure 16.

**Figure 17:**
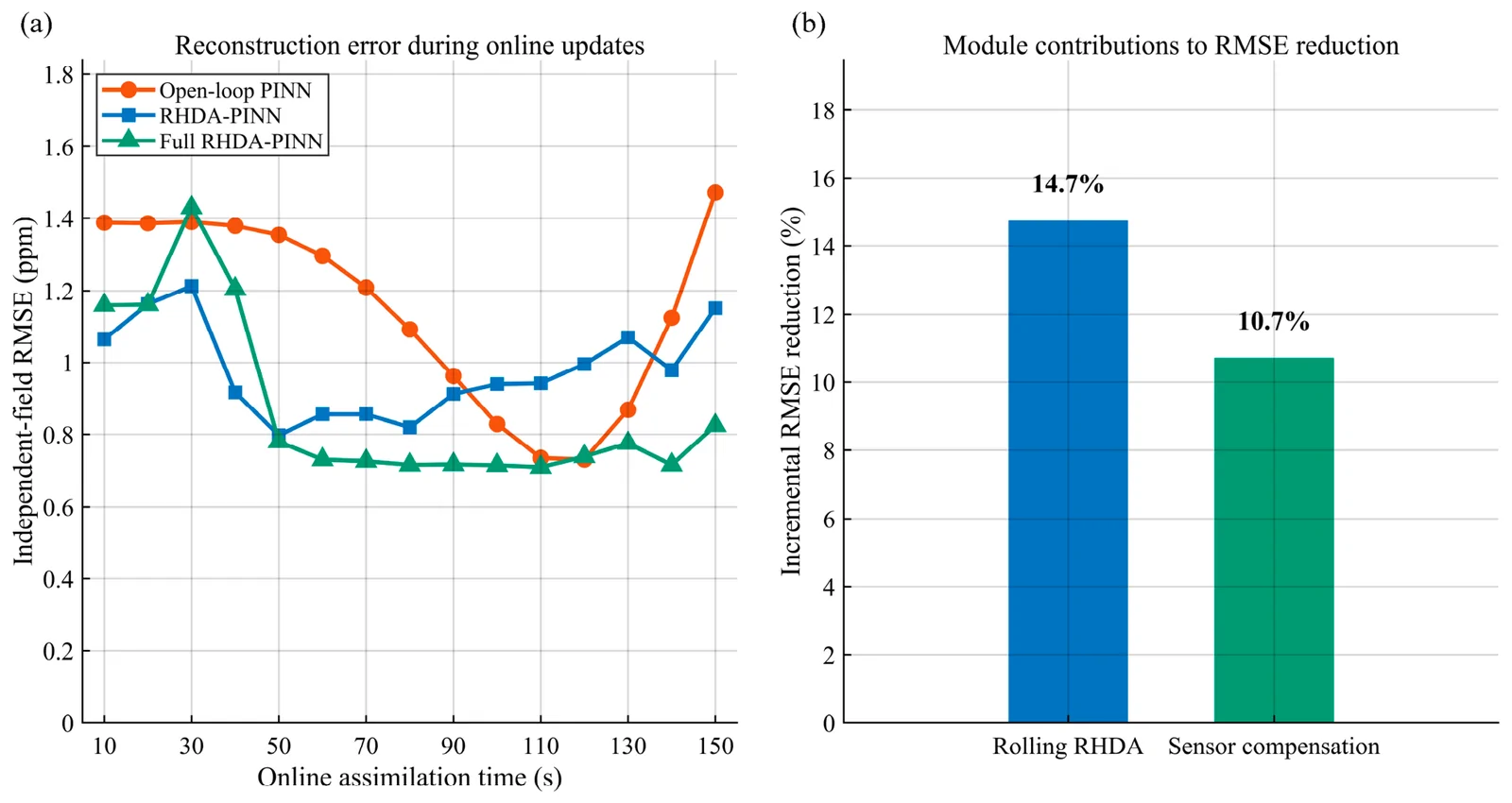
Ablation of the reconstruction framework using COMSOL-derived virtual observations. (**a**) Independent-field RMSE during online updates; (**b**) RMSE reductions produced by RHDA and sensor-dynamic compensation.

To isolate the effects of the different methods on reconstruction performance, all three approaches employed the same offline-pretrained PINN, network architecture, and evaluation dataset. The results show that the open-loop PINN, the RHDA-PINN assimilating lagged COMSOL-derived virtual node observations, and the full RHDA-PINN incorporating sensor dynamic compensation achieved mean independent-field RMSE values of 1.1477, 0.9786, and 0.8736 ppm, respectively. Compared with the open-loop PINN, rolling RHDA reduced the RMSE by 14.74%, while sensor dynamic compensation provided a further reduction of 10.72%. These results demonstrate that periodic rolling assimilation can effectively correct biases in the offline prior, whereas sensor dynamic compensation mitigates the influence of sensor response lag on the assimilated observations. Consequently, the full RHDA-PINN achieves the highest average reconstruction accuracy over the entire evaluation period, as shown in Figure 17.

All computations were performed in MATLAB R2023b on a workstation equipped with an NVIDIA GeForce RTX 3070 GPU (8 GB memory). Offline pre-training used 60,000 supervised points for 500 epochs with a mini-batch size of 8192, while 6000 PDE collocation points and 1200 boundary points were dynamically sampled at each optimizer update. The complete 500-epoch pre-training required 478.692 s (7.978 min). Inference over 20,000 validation points required 0.325 s. During RHDA, each 10 s assimilation window used 10 Adam update steps and required 1.158 s on average, corresponding to 11.58% of the update interval. Offline pre-training was completed before deployment and was therefore not included in the online latency.

## 4. Dynamic Reconstruction

Subsequently, multidimensional validation of the PINN-based gas-diffusion model was conducted. First, the global fitting accuracy of the static pre-trained model was evaluated. Subsequently, the analysis focused on the error suppression capability of the RHDA mechanism under dynamic disturbances and long-term monitoring. Concurrently, the effects of different front-end sensing material compositions on algorithmic accuracy were quantitatively considered, elucidating the critical role of high-sensitivity observational data in real-time model rectification. Finally, temporal extrapolation and 3D super-resolution reconstruction are evaluated within the fixed chamber geometry used in this study.

### 4.1. Global Accuracy Validation of the Static Pre-Trained Model

**Figure 18:**
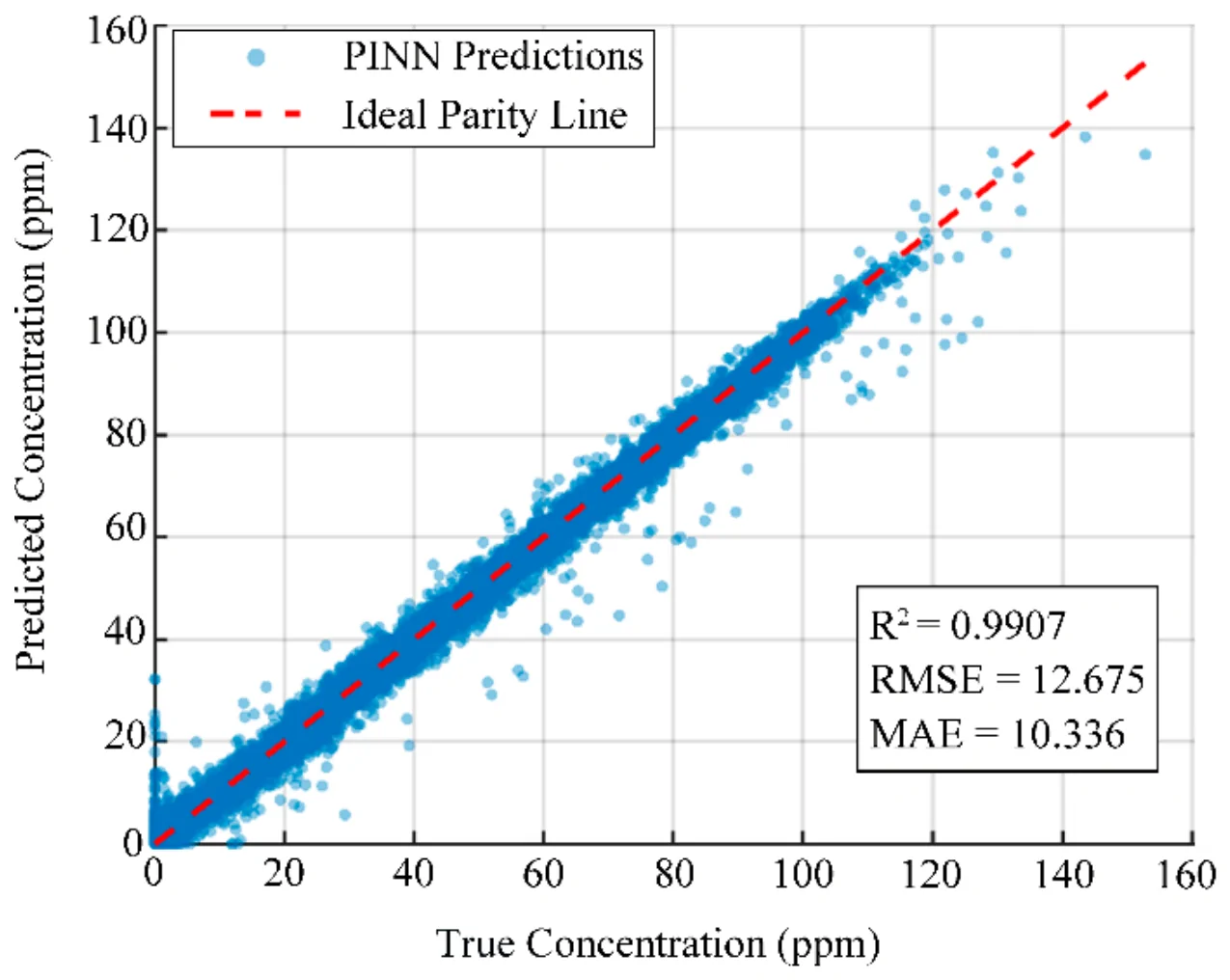
Parity between PINN-predicted concentrations and COMSOL reference values over the evaluated spatiotemporal field.

To comprehensively evaluate the surrogate predictive capability of the PINN for the gas diffusion process within a confined space, Figure 18 presents the global parity plot of the predicted concentration versus the COMSOL simulation results. The comparison evaluates how well the data-informed PINN approximates the COMSOL reference concentration field while using a reduced-order diffusion constraint.

As shown in Figure 18, the predicted points are closely and symmetrically distributed on both sides of the ideal parity line (red dashed line). Notably, the model exhibits no distinct systematic underfitting or overfitting phenomena either in the low-concentration regime (~0–40 ppm), which dominates the background during the early stages of gas diffusion, or in the high-concentration core regime (>100 ppm) resulting from thermal buoyancy accumulation and boundary spreading. Although extremely minor vertical dispersion is evident near the ultra-low concentration region (~0 ppm), this was primarily attributed to the fitting tolerance of the PINN activation function to the hard-zero boundary, which does not substantially affect the topological reconstruction of the overall macroscopic diffusion flow field.

In terms of statistical quantitative indicators, the current model demonstrates extremely high predictive confidence. Specifically, its coefficient of determination (R^2^) reaches 0.9907, indicating that the neural network successfully explains >99% of the spatiotemporal variability of the concentration field. Simultaneously, its global RMSE and mean absolute error are effectively controlled at 12.675 and 10.336 ppm, respectively. Compared with the local peak surge concentration of ~160 ppm inside the chamber, an absolute error of this magnitude indicates that the model maintains high numerical reconstruction accuracy throughout the entire 3D inference space, providing a reliable foundation for subsequent closed-loop evolution and digital-twin applications.

### 4.2. Effect of Active Sensor Number on Reconstruction Accuracy

A COMSOL-based virtual observation-anchor study was performed to assess the effect of sensor number. The same 3 × 3 × 3 candidate layout was progressively refined as 2 × 2 × 2 (Ns = 8), 3 × 2 × 2 (Ns = 12), 3 × 3 × 2 (Ns = 18), and 3 × 3 × 3 (Ns = 27). All cases used the same fixed parameters and were evaluated from t = 10 to 150 s at 10 s intervals.

**Figure 19:**
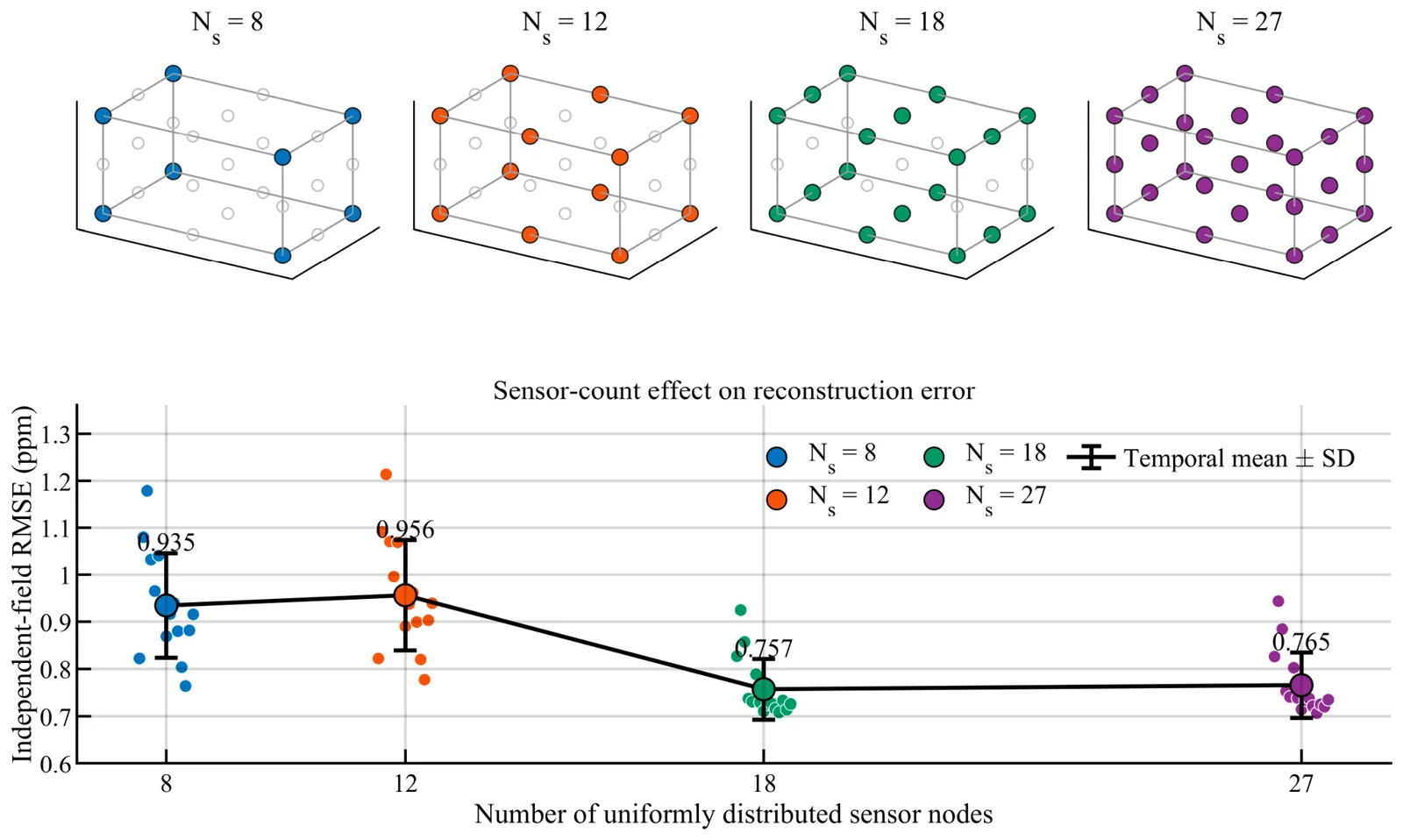
Uniform layouts with 8, 12, 18, and 27 observation nodes and the corresponding independent-field RMSE.

As shown in Figure 19, the temporal mean ± SD independent-field RMSE values were 0.935 ± 0.111, 0.956 ± 0.117, 0.757 ± 0.064, and 0.765 ± 0.069 ppm for N_s = 8, 12, 18, and 27, respectively. Increasing the node count from 12 to 18 reduced the mean RMSE by 20.9%. The difference between the 18-node and 27-node layouts was only 1.1%, indicating diminishing accuracy gains beyond 18 nodes. Although the 18-node layout achieved the lowest mean RMSE in this specific scenario, the uniform 27-node layout provided complete volumetric coverage and greater redundancy against node failures. Therefore, 27 nodes were selected as an engineering balance among reconstruction accuracy, three-dimensional observability, and robustness.

A random sensor-node failure study was conducted using a fixed 27-node reference configuration, common COMSOL time slices, independent-field evaluation points, a deterministic open-loop prior, a reconstruction kernel, and RMSE definition across all failure levels. Within this Monte Carlo masking protocol, the no-failure reference RMSE was 0.694 ppm. Reconstruction accuracy degraded gradually as nodes were removed, with pronounced increases in error and uncertainty occurring only under severe node loss. These results indicate that the framework is robust to limited and moderate random sensor failures. The complete protocol, statistics for 40 random patterns, validation results, and 800 dpi figure are provided in the Consolidated Supporting Information (Section S2 and Supplementary Figure S2).

### 4.3. Dynamic Robustness and Long-Term Accuracy Analysis of the Closed-Loop PINN Based on Rolling Horizon Data Assimilation

**Figure 20:**
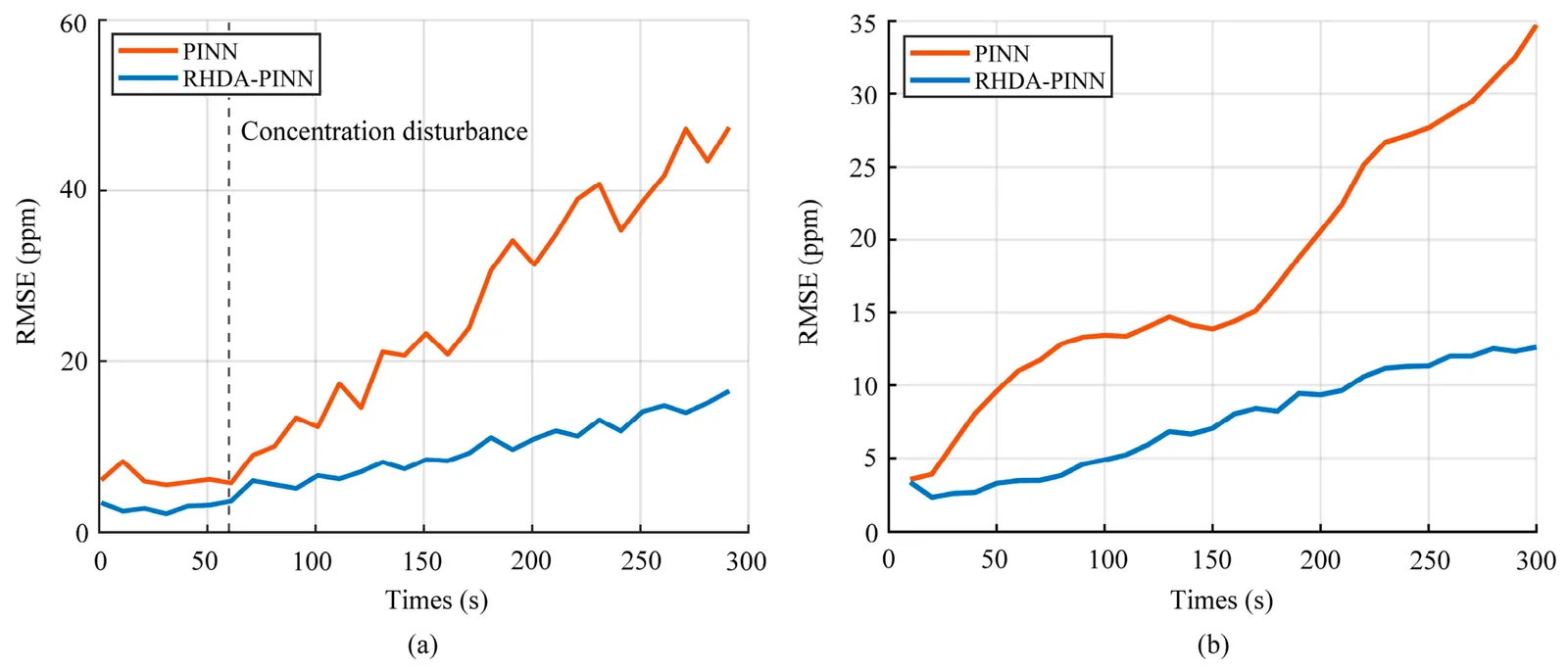
RMSE evolution of the open-loop PINN and RHDA-PINN. (**a**) Response to a concentration disturbance introduced at 60 s; (**b**) prediction error during 300 s continuous monitoring.

The primary advantage of the closed-loop fine-tuning PINN incorporating a rolling horizon online assimilation mechanism (RHDA-PINN) lies in its adaptive capability in complex and dynamic environments. Figure 20 compares the temporal evolution trends of the global RMSE between the open-loop pre-trained PINN and the RHDA-PINN under different test scenarios.

Specifically, Figure 20a evaluates the system-level robustness of the model against sudden concentration disturbances. In this case, a transient concentration disturbance was simulated at *t* = 60 s via the heated evaporation of an additional droplet of 1% diluted ammonia water introduced into the chamber, thereby simulating an instantaneous increase in the strength of the pollution source in a real monitoring scenario. During the steady diffusion period (0–60 s), the RMSE of both models remained at a low baseline level (i.e., below ~8 ppm). Following the introduction of the concentration surge disturbance at *t* = 60 s, the evolution trajectories of the two models diverged significantly. Due to the lack of real-time environmental perception and dynamic feedback mechanisms, the open-loop PINN failed to correct the fundamental model deviation caused by the sudden environmental change. Consequently, its prediction error accumulated continuously through the time integration, exhibiting severe structural divergence and surging to ~48 ppm by the end of the evolution period. In contrast, using the online data-assimilation mechanism, the RHDA-PINN assimilated the limited COMSOL-derived virtual node observations at each update to rapidly correct the underlying network weights. Even in the presence of more pronounced sudden changes in the internal flow field, the global RMSE exhibited only a gradual increase following the disturbance and remained stable at ~16 ppm, demonstrating excellent resistance to dynamic interference.

Figure 20b further illustrates the baseline error accumulation of the inference models over a 300 s continuous monitoring period. The open-loop PINN without real-time data correction exhibited a significant error accumulation effect, wherein its RMSE increased monotonically from the early stage of simulation, ultimately reaching a maximum of ~35 ppm at 300 s. This indicates that a purely theory-driven physical model struggles to autonomously suppress the progressive amplification of computational residuals over long integration times. Conversely, the RHDA-based closed-loop system demonstrated superior error constraint capability. Throughout the monitoring cycle, the RMSE of the RHDA-PINN was effectively suppressed, stabilizing at ~13 ppm at 300 s. Thus, by periodically incorporating COMSOL-derived virtual node feedback during the controlled evaluation, RHDA suppressed long-term error accumulation relative to the open-loop PINN. In deployment, the same assimilation interface is designed to accept calibrated feedback from the physical sensor array.

### 4.4. Evaluation of 3D Spatial Field Reconstruction Accuracy for the Closed-Loop PINN Framework

**Figure 21:**
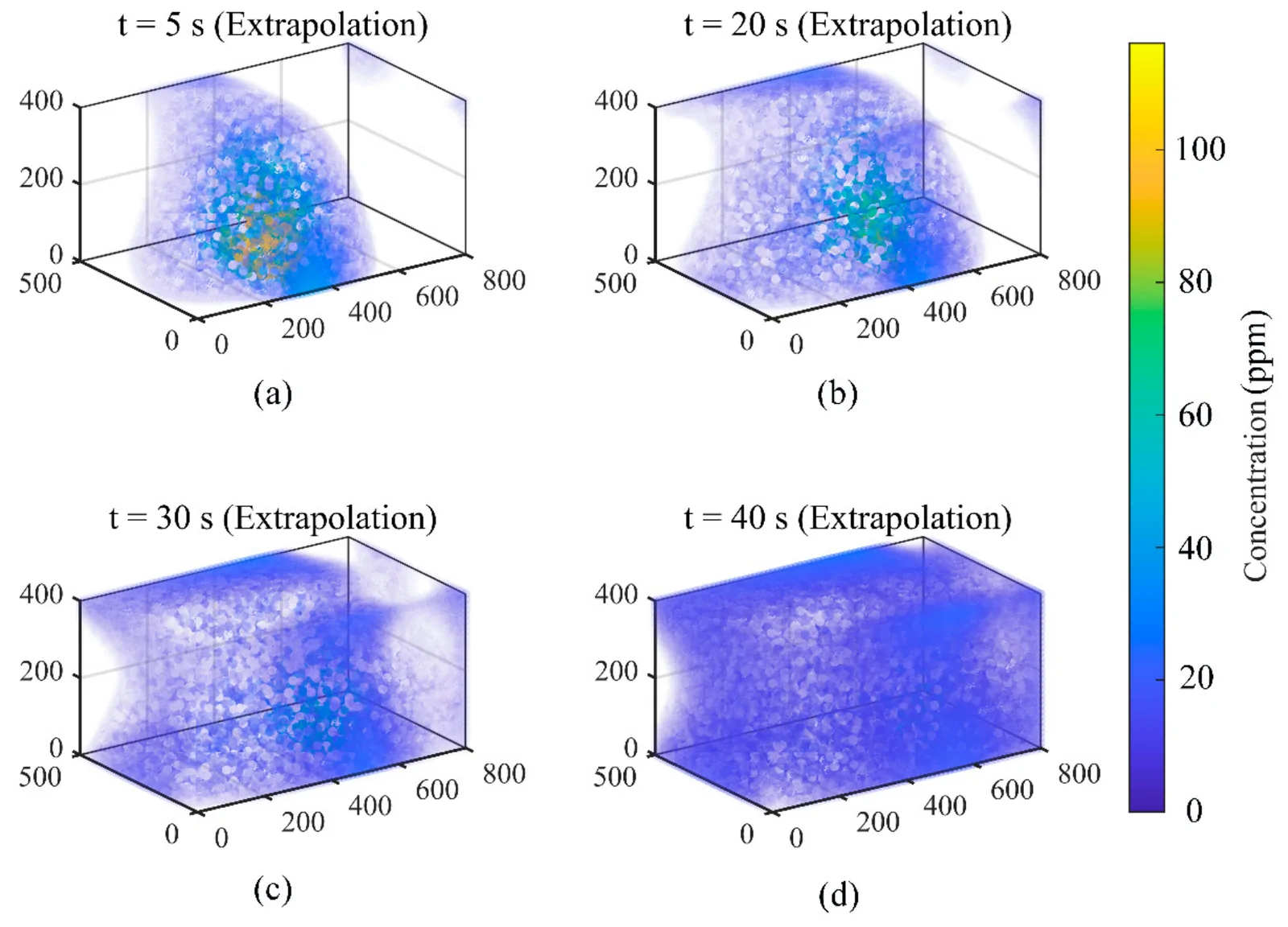
Three-dimensional RHDA-PINN extrapolation of the NH_3_ concentration field at different times: (**a**) at t = 5 s, a localized high-concentration region is observed near the release area, with a peak concentration exceeding 100 ppm; (**b**) at t = 20 s, the NH_3_ plume expands within the chamber while its peak concentration gradually decreases; (**c**) at t = 30 s, continued diffusion and redistribution produce a broader and more dispersed concentration field; and (**d**) at t = 40 s, NH_3_ is distributed over most of the chamber, with an overall lower and more uniform concentration. The common color scale represents the NH_3_ concentration in ppm.

To intuitively evaluate the 3D visualization performance of the gas diffusion model, Figure 21 presents the 3D particle cloud diagrams corresponding to *t* = 5, 20, 30, and 40 s. As shown, the RHDA-PINN demonstrates a super-resolution visualization capability after relaxing the constraints of traditional finite element meshes. From the perspective of macroscopic fluid morphology, even in the extrapolation stage beyond the training time domain, the 3D particle clouds reconstructed by the RHDA-PINN effectively preserve the dynamic topological evolution patterns. At *t* = 5 s, the model accurately captures the cluster-like high-concentration accumulation core during the initial stage of gas release, rather than a single vertical plume. Additionally, at *t* = 20 and 30 s, the PINN reproduces the contour where the gas front contacts the chamber boundary, undergoes momentum deflection, and extensively spreads and propagates along the inner wall surfaces. By 40 s, the gas is fully dispersed, generally presenting a dark blue tone (i.e., a low concentration) and indicating a trend toward a globally homogenized state. The results indicate that the reduced-order diffusion prior preserves the large-scale concentration evolution, while the supervised data and RHDA updates recover part of the convection- and buoyancy-related structures.

**Figure 22:**
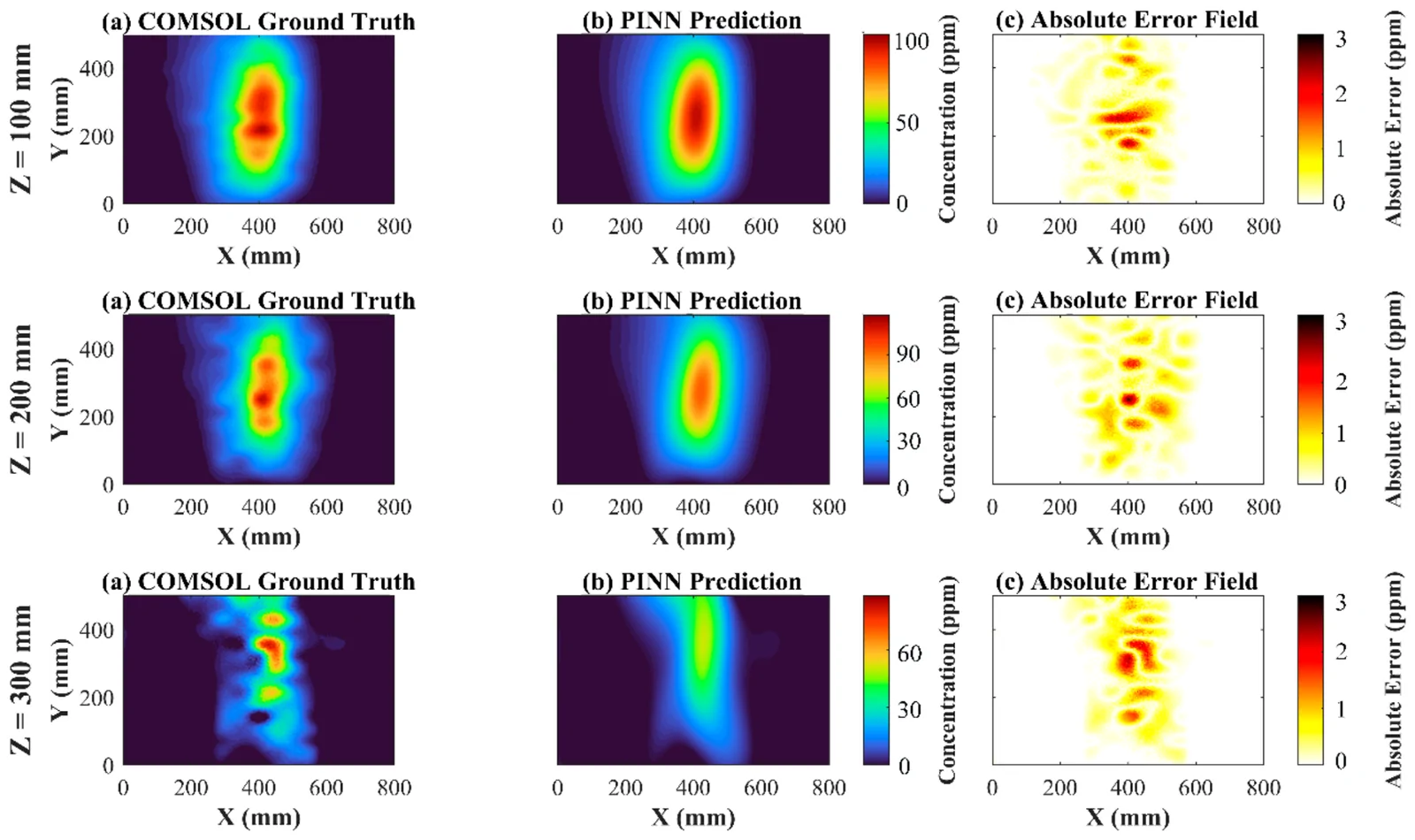
COMSOL reference fields, RHDA-PINN reconstructions, and absolute errors at Z = 100, 200, and 300 mm. The labels (**a**), (**b**), and (**c**) denote the columns: COMSOL ground truth, RHDA-PINN prediction, and absolute error field, respectively, and apply to all three rows. The top, middle, and bottom rows correspond to Z = 100, 200, and 300 mm, respectively.

Figure 22 compares 2D orthogonal slices at three different heights (Z = 100, 200, and 300 mm) within the chamber to verify the spatial reconstruction fidelity of the RHDA-PINN model. As shown in Figure 22b, the RHDA-PINN prediction field not only accurately captures the central high-concentration core region but also clearly reproduces the spatial stratification characteristics associated with the upward transport of NH_3_, which is driven by thermal buoyancy (i.e., as the height increases, the core peak concentration gradually decreases from ~100 ppm at Z = 100 mm to ~60 ppm at Z = 300 mm).

Quantitative error analysis indicates that the absolute error distribution of the diffusion model is closely related to the complexity of the local flow field and the concentration gradients. Since real fluid diffusion is accompanied by weak convective disturbances, such as the edge folds and irregular shapes observed in the COMSOL ground-truth field (Figure 22a), and since the RHDA-PINN model exhibits a certain manifold smoothing effect when fitting partial differential equation residuals, the errors are predominantly concentrated in the plume core region and local regions with sharp morphological variations. Across all height slices, the global maximum absolute error is effectively controlled at ~3 ppm, indicating that the network approximation error does not substantially affect the dynamic inference of the macroscopic flow field.

### 4.5. Pure Physical Extrapolation and Error Robustness Analysis of Long-Term Evolution

**Figure 23:**
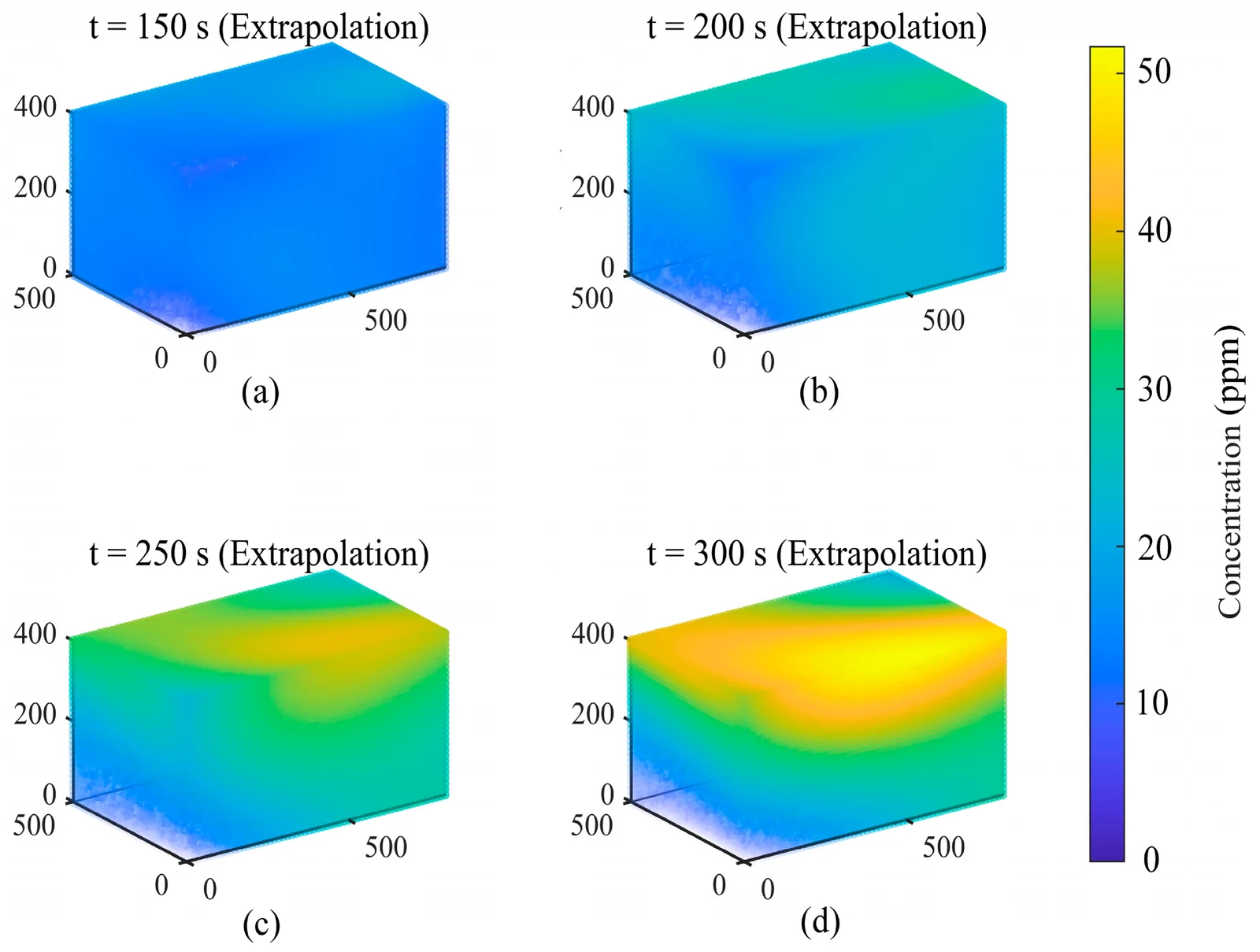
Three-dimensional RHDA-PINN extrapolation of the NH_3_ concentration field beyond the training time domain: (**a**) t = 150 s, showing the early extrapolation state with a relatively low concentration; (**b**) t = 200 s, showing progressive gas accumulation and upward transport; (**c**) t = 250 s, showing the development of vertical concentration stratification; and (**d**) t = 300 s, showing pronounced NH_3_ enrichment in the upper region of the confined chamber, with a peak concentration of approximately 50 ppm. The shared color bar represents the NH_3_ concentration in ppm.

Figure 23 presents the 3D physical extrapolation results of the RHDA-PINN model beyond the training time domain (t ≥ 150 s). This evolution sequence (t = 150, 200, 250, and 300 s) clearly reproduces the long-term dispersion and saturated accumulation process of NH_3_ within the confined space. Benefiting from the strong constraint of the Neumann no-flux boundary condition in the loss function, the flow field does not exhibit non-physical boundary leakage or numerical divergence. As the initial thermal convective kinetic energy is gradually dissipated, the macroscopic migration of the gas smoothly transitions to the molecular diffusion stage dominated by Fick’s law. Observing Figure 23a–d, it is evident that the overall gas concentration gradually increases over time and exhibits a significant vertical spatial stratification phenomenon. Specifically, by t = 300 s, a higher concentration of NH_3_ has accumulated in the upper space of the chamber (bright yellow tone, peak concentration ≈ 50 ppm), while the concentrations in the middle and lower layers remain relatively low. This extrapolated evolution process conforms to the dynamic gas laws under the influence of density differences and residual buoyancy, demonstrating reliable long-term temporal extrapolation within the tested chamber geometry.

**Figure 24:**
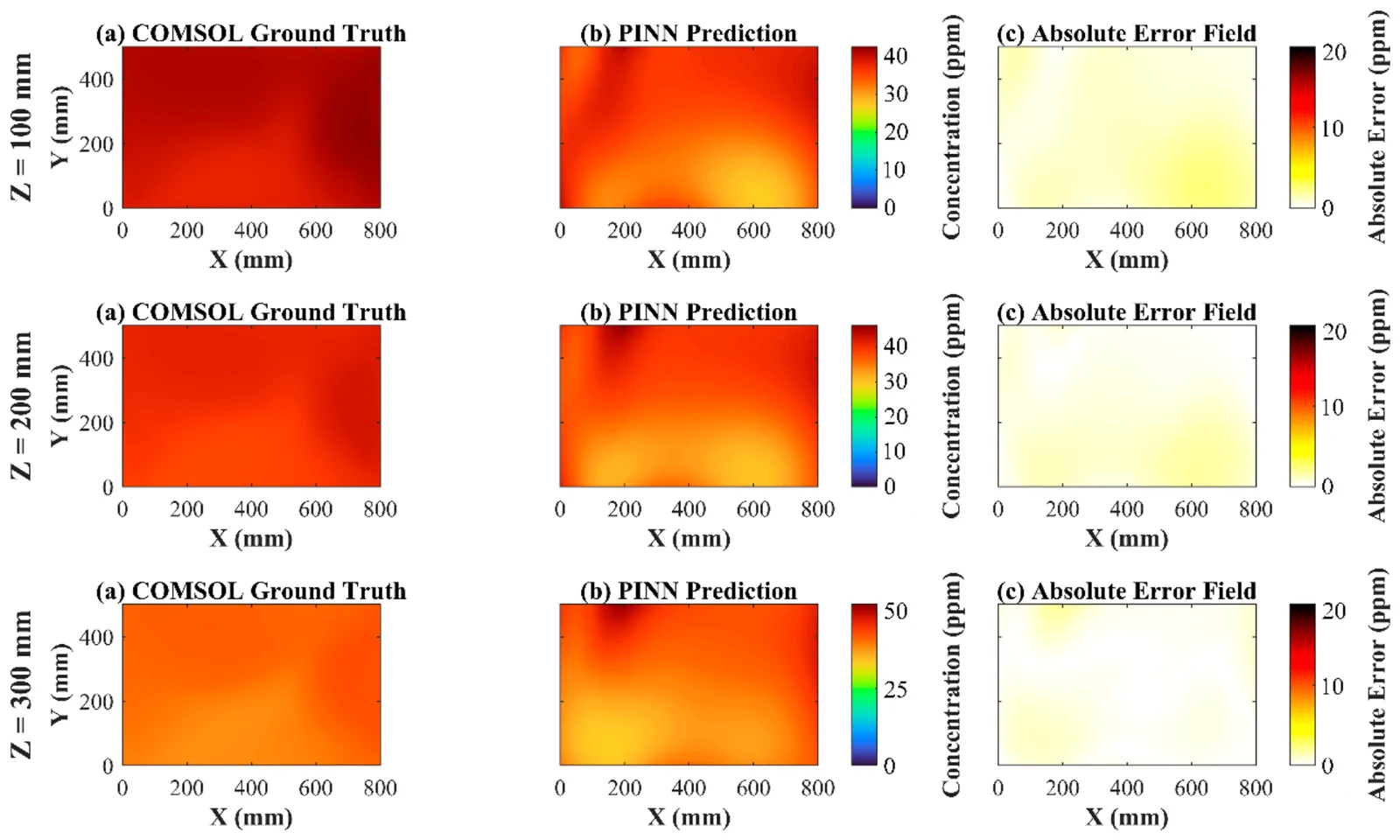
COMSOL reference fields, PINN predictions, and absolute errors at Z = 100, 200, and 300 mm in the t = 240 s extrapolation case. The labels (**a**), (**b**), and (**c**) denote the columns: COMSOL ground truth, PINN prediction, and absolute error field, respectively, and apply to all three rows. The top, middle, and bottom rows correspond to Z = 100, 200, and 300 mm, respectively.

Figure 24 shows the reconstruction results of the 2D orthogonal slices taken from the long-term physical extrapolation of the RHDA-PINN model beyond the training time domain (taking t = 240 s as an example). By comparing the concentration distributions at three heights (Z = 100, 200, and 300 mm), it can be seen that during the phase entirely decoupled from offline data-driven supervision, the RHDA-PINN prediction field (Figure 24b) accurately replicates the macroscopic topological structure of the COMSOL ground-truth field (Figure 24a). Specifically, the model effectively reproduces the dispersion contour of the gas in the horizontal direction while also capturing the vertical stratification characteristics, where the background concentration significantly increases with height. In particular, the Z = 300 mm slice exhibits a distinctly higher overall concentration than the lower slices, with a local peak of ~50 ppm, demonstrating a high degree of fluid-dynamic consistency. Quantitative error analysis (Figure 24c) further confirms the reliability of the long-term model inference. In all observed slices, the global absolute error remains low, generally falling below 10 ppm for most regions. Additionally, the absolute error exhibits a spatially random distribution without the accumulation of structural deviations, successfully avoiding the temporal divergence that is prone to occur in traditional purely data-driven models during long-term prediction. This indicates that the network possesses excellent error-robust convergence capability during extrapolated evolution.

Despite employing a reduced-order Fick’s diffusion law as the underlying physical constraint, the RHDA-PINN system accurately reconstructs the vertical NH_3_ plume with prominent convective characteristics during the initial leakage stage [50]. This behavior was primarily attributed to the RHDA mechanism. In the controlled reconstruction evaluation, COMSOL-derived virtual observations at the 27 predefined node locations served as assimilation anchors during convection-dominated stages and guided rolling updates of the network weights. This rolling calibration compensated for transport components not explicitly represented by the reduced-order PDE constraint. Within the proposed system architecture, the same RHDA interface is used to assimilate observations supplied by the physical 27-channel sensing system, thereby connecting the sensing hardware with the reconstruction model. Moreover, the system exhibits superior spatiotemporal generalization in long-term inference, achieving high-fidelity 3D reconstruction of the NH_3_ diffusion field within the confined space.

Two controlled cross-scenario transfer tests were conducted. In Scenario A, the chamber was enlarged from 800 mm × 500 mm × 400 mm to 1000 mm × 600 mm × 500 mm and a low ventilation velocity of 4.0 mm s^−1^ was introduced in the x direction. Scenario B retained the original chamber dimensions but relocated the leakage source from approximately (390, 250, 0) mm to (620, 150, 0) mm. The normalized geometry, boundary conditions, velocity/source terms, and collocation domain were redefined, and the same 4 × 64 RHDA-PINN underwent limited transfer refinement before evaluation at unseen times of 130–150 s using independent field points that excluded the sensing nodes and the leakage-source neighborhood. Scenario A achieved mean RMSE, NRMSE, and NMAE values of 0.0380 ppm, 2.41%, and 1.32%, respectively, with full-field/active-plume R^2^ values of 0.896/0.926. Scenario B achieved corresponding values of 0.0662 ppm, 1.83%, and 0.99%, with R^2^ values of 0.895/0.915. After 0.015 ppm observation noise was introduced, RHDA updates increased the mean RMSE relative to the transferred prior by only 4.47% and 1.67%, respectively, without unstable amplification. These results support the transferability of the workflow after physical reconfiguration and limited transfer refinement rather than unconditional zero-shot transfer of fixed weights to arbitrary rooms. Detailed results are provided in Supplementary Figure S3 and the Consolidated Supporting Information.

## 5. Conclusions

This study develops a closed-loop architecture for three-dimensional NH_3_ diffusion reconstruction in confined spaces by integrating the fabricated PPy/Graphene/WO_3_ sensor array with an RHDA-compensated physics-informed diffusion model. The ternary sensing material consists of 0D PPy nanospheres, 1D WO_3_ nanorods, and 2D graphene nanosheets and detects NH_3_ at room temperature. Its calculated LOD is 0.0470 ppm, and supplementary dynamic experiments directly validate a response at 0.1 ppm. The 27-channel platform provides spatially distributed observations, while Fick’s second law and Neumann boundary conditions constrain the mesh-free PINN. RHDA assimilates dynamically compensated observations every 10 s to correct the diffusion model and limit open-loop drift caused by disturbances and model mismatch. Relative to the open-loop PINN, RHDA improves disturbance tracking and long-term reconstruction while maintaining a low online computational cost. Sensor-count and random-failure analyses showed that the 18-node layout achieved the lowest mean RMSE in the tested scenario (0.757 ppm), whereas the 27-node layout produced a comparable RMSE (0.765 ppm; a difference of only 1.1%) and was selected because its uniform arrangement provides more complete volumetric coverage and greater redundancy against node failures. After physical reconfiguration and limited transfer refinement, the enlarged ventilated-chamber and relocated-source scenarios achieved mean NRMSE values of 2.41% and 1.83%, respectively, supporting the transfer of the workflow across the tested scenarios. Coupling the distributed sensor array to the diffusion model through RHDA allows rolling sensor feedback to compensate for the physics-constrained model and converts discrete measurements into continuous three-dimensional field estimates, providing a new basis for spatially resolved ammonia monitoring.

## Data Availability

The original contributions presented in this study are included in the article/Supplementary Materials. Further inquiries can be directed to the corresponding author.

## Supplementary Materials

The following supporting information can be downloaded at: https://www.mdpi.com/article/10.3390/polym18172149/s1, Figure S1: Dynamic response of the PPy/Graphene/WO3-3 sensor to 100 ppb, 500 ppb, 1 ppm, and 5 ppm NH3; Figure S2: Independent-field reconstruction RMSE under random sensor-node failures; Figure S3: Reference fields and RHDA-PINN predictions at t = 150 s for the enlarged ventilated chamber and the relocated-source scenario; Figure S4: Dynamic responses to NH3, CO2, CO, NO, H2S, acetone, ethanol, and formaldehyde at 50 ppm; Figure S5: COMSOL-simulated spatiotemporal evolution of NH3 in the confined chamber; Figure S6: Device-to-device reproducibility of 27 PPy/Graphene/WO3-3 sensors at 30 ppm NH3; Figure S7: Agreement between the 27-channel sensor input and COMSOL node references, the temporal response of representative channel 05, input RMSE across the 27 channels, and independent-field RMSE for the open-loop PINN and sensor-observation-driven RHDA.

## Abbreviations

The following abbreviations are used in this manuscript:

PINN	Physics-informed neural network
PPy	Polypyrrole
0D	Zero-dimensional
1D	One-dimensional
2D	Two-dimensional
3D	Three-dimensional
MOS	Metal oxide semiconductor
CFD	Computational fluid dyamics
SNR	Signal-to-noise ratio
RHDA	Rolling horizon data assimilation
RMSE	Root mean square error
SEM	Scanning electron microscopy
XRD	X-ray diffractometry
FTIR	Fourier transform infrared
APS	Ammonium persulfate
EDS	Energy-dispersive X-ray spectroscopy
LOD	Limit of detection
LHS	Latin hypercube sampling
PDE	Partial differential equation
