# Supplementary material for: Polypyrrole/Graphene/WO3 Ternary Nanocomposite Sensor Arrays for 3D Ammonia Leakage Reconstruction via Rolling Horizon Data-Assimilated PINNs
Source: Polymers (Basel). 2026 Sep 2;18(17):2149. doi: 10.3390/polym18172149 (PMC13568352; doi:10.3390/polym18172149)
Supplement: Supplementary file 1 [file polymers-18-02149-s001.zip › polymers-4490822-supplementary.pdf]

Supporting Information

Polypyrrole/Graphene/WO_3 Ternary Nanocomposite Sensor Arrays for 3D Ammonia Leakage Reconstruction via Rolling Horizon Data-Assimilated PINNs

Haodong Niu [1,2], Yunbo Shi [1,2,*], Kuo Zhao [1,2], Jinzhou Liu [1,2], Xiaohui Yang [1,2] and Canda Zheng [1,2]

1 School of Measurement and Control Technology and Communication Engineering, Harbin University of Science and Technology, Harbin 150080, China; 2010600001@stu.hrbust.edu.cn (H.N.); iridescen7@163.com (K.Z.); liujinzhou0709@outlook.com (J.L.); 420610159@stu.hrbust.edu.cn (X.Y.); 2410603075@stu.hrbust.edu.cn (C.Z.)

2 China Higher Educational Key Laboratory for Measuring & Control Technology and Instrumentations of Heilongjiang Province, School of Measurement-Control Technology and Communications Engineering, Harbin University of Science and Technology, Harbin 150080, China

* Correspondence: shiyunbo@hrbust.edu.cn

Scope. This Supporting Information contains seven supplementary analyses that are not reproduced as figures in the main manuscript. These analyses cover low-concentration dynamic validation, random sensor-node failure robustness, cross-scenario transfer validation, gas-specific dynamic response traces, COMSOL simulation and dataset construction, and device-to-device reproducibility of the 27-sensor array. Section S7 further evaluates real-time sensor-data input to Sensor-observation-driven RHDA using a time-controlled replay of experimentally recorded observations from the fabricated 27-channel sensor array.

S1. Low-Concentration Dynamic-Response Validation

The calculated limit of detection was obtained using LOD = $3\sigma_{blank}/S$, where σ_{blank} = 0.0140% is the standard deviation of the stable air-baseline response and S = 0.8962% ppm^{-1} is the slope of the 5–50 ppm calibration curve. This gives 0.0469 ppm, reported as 0.0470 ppm. To provide direct low-concentration evidence, the PPy/Graphene/WO_3-3 sensor was additionally exposed to 100 ppb, 500 ppb, 1 ppm, and 5 ppm NH_3 under the same room-temperature protocol.

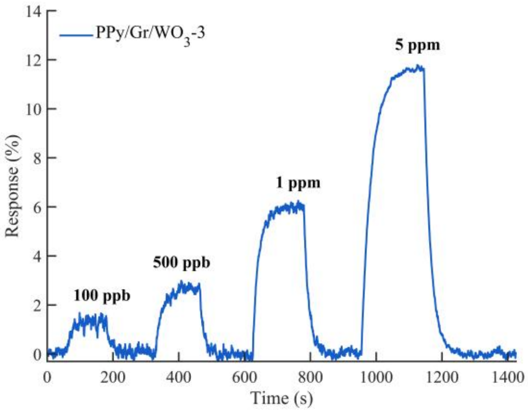

Supplementary Figure S1. Dynamic response of the PPy/Graphene/WO_3-3 sensor to 100 ppb, 500 ppb, 1 ppm, and 5 ppm NH_3.

The corresponding peak responses were approximately 1.57%, 2.87%, 6.14%, and 11.69%, respectively. The response at 100 ppb exceeded the baseline fluctuation and returned close to baseline after NH_3 removal. Accordingly, 0.0470 ppm is retained as the calculated LOD, while 0.1 ppm is the lowest concentration directly validated experimentally.

S2. Reconstruction Robustness under Random Sensor-Node Failures

The failure analysis used a fixed 27-node reference configuration, COMSOL time slices from 30 to 150 s at 10 s intervals, array-bounded independent evaluation points, a deterministic open-loop prior, a kernel length of 0.32, a ridge parameter of 0.12, and a common RMSE definition across all failure levels. Starting from the 27-node layout, 0, 3, 6, 9, 12, 15, 18, and 21 nodes were removed at random. Forty independent failure-location patterns were evaluated at each nonzero failure level. Because the purpose of this Monte Carlo test is to quantify sensitivity to random node removal, all changes are interpreted relative to the zero-failure reference within this fixed masking protocol.

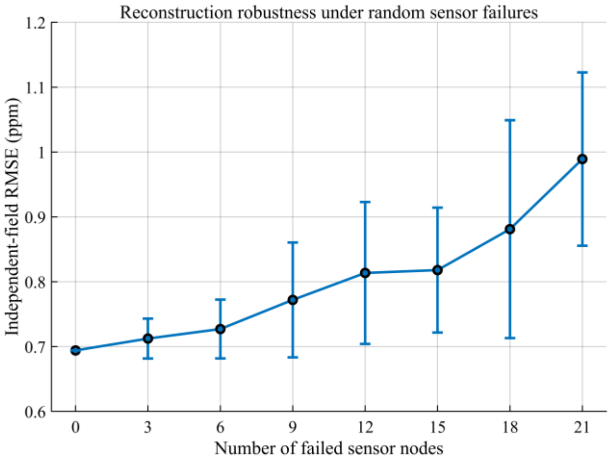

Supplementary Figure S2. Independent-field reconstruction RMSE under random sensor-node failures. Error bars denote the standard deviation over 40 random failure-location patterns.

The zero-failure RMSE was 0.694 ppm. With 3 and 6 failed nodes, the RMSE values were 0.712 ± 0.031 and 0.727 ± 0.045 ppm, corresponding to increases of 2.6% and 4.7%. With 9 and 12 failed nodes, the RMSE increased to 0.772 ± 0.089 and 0.814 ± 0.109 ppm. When 21 nodes failed and only six remained active, the RMSE reached 0.989 ± 0.134 ppm, 42.5% above the zero-failure reference. The results indicate modest degradation under limited or moderate random failures and pronounced uncertainty growth only under severe node loss.

S3. Cross-Scenario Transfer Validation

Two controlled scenarios were used to test transfer beyond the original 800 × 500 × 400 mm chamber. Scenario A enlarged the chamber to 1000 × 600 × 500 mm and introduced a weak x-directed ventilation velocity of 4.0 mm s^{-1}. Scenario B retained the original chamber size but relocated the leakage source from approximately (390, 250, 0) mm to (620, 150, 0) mm. The physical domain, boundary conditions, velocity/source terms, and collocation region were redefined. The same 4 × 64 RHDA-PINN underwent limited transfer refinement and was evaluated at unseen times from 130 to 150 s using independent field points that excluded the sensing nodes and leakage-source neighborhood.

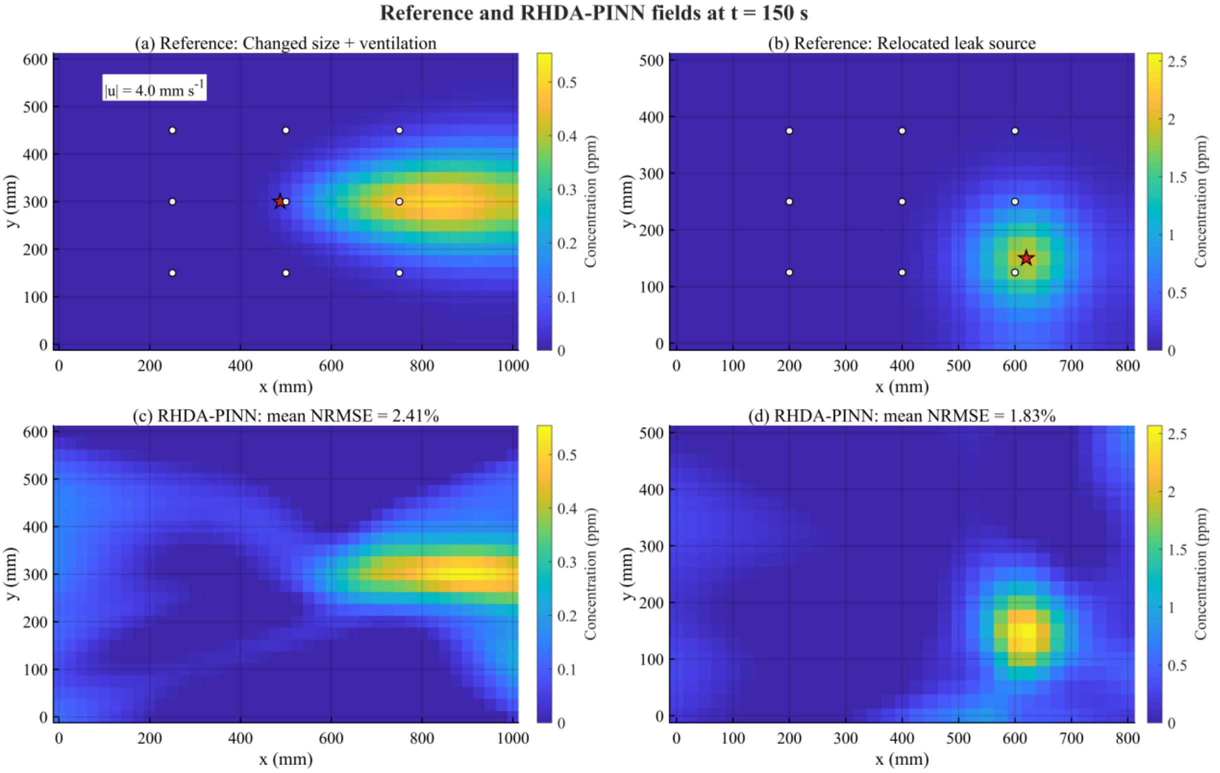

Supplementary Figure S3. Reference fields and RHDA-PINN predictions at t = 150 s for (a, c) the enlarged ventilated chamber and (b, d) the relocated-source scenario. Red stars indicate leakage-source locations; white circles indicate projected sensor columns.

Scenario	Physical modification	Mean RMSE / NRMSE / NMAE	R^2 (full/active); noise-induced ΔRMSE
A	1000 × 600 × 500 mm chamber; x-directed ventilation of 4.0 mm s^{-1}	0.0380 ppm / 2.41% / 1.32%	0.896 / 0.926; +4.47%
B	Original chamber; source relocated to approximately (620, 150, 0) mm	0.0662 ppm / 1.83% / 0.99%	0.895 / 0.915; +1.67%

After 0.015 ppm observation noise was introduced, the conservative RHDA updates increased the mean RMSE relative to the transferred prior by only 4.47% and 1.67% in Scenarios A and B, respectively, without unstable amplification. These controlled results support transfer of the workflow after physical reconfiguration and limited refinement; they do not imply unconditional zero-shot portability of fixed weights to arbitrary rooms.

S4. Gas-Specific Dynamic Response Traces

To complement the static 50 ppm selectivity comparison, the dynamic traces were evaluated using the resistance-change ratio. Here, R_a is the stable resistance in air immediately before exposure and R_g is the resistance under the target gas. The same 120–300 s exposure window was used for all gases, enabling direct comparison of amplitude, noise, reversibility, and recovery behavior.

$$Response\ (\%) = |R_g - R_a|/R_a \times 100\%$$

The peak responses were approximately 50%, 5%, 9%, 15%, 14%, 11%, 6%, and 6% for NH_3, CO_2, CO, NO, H_2S, acetone, ethanol, and formaldehyde, respectively. The NH_3 response was therefore at least 3.3 times the largest interfering-gas response. The gas-dependent noise, residual baseline offsets, and incomplete or slower recovery visible in several interfering-gas traces provide a dynamic complement to the static selectivity values.

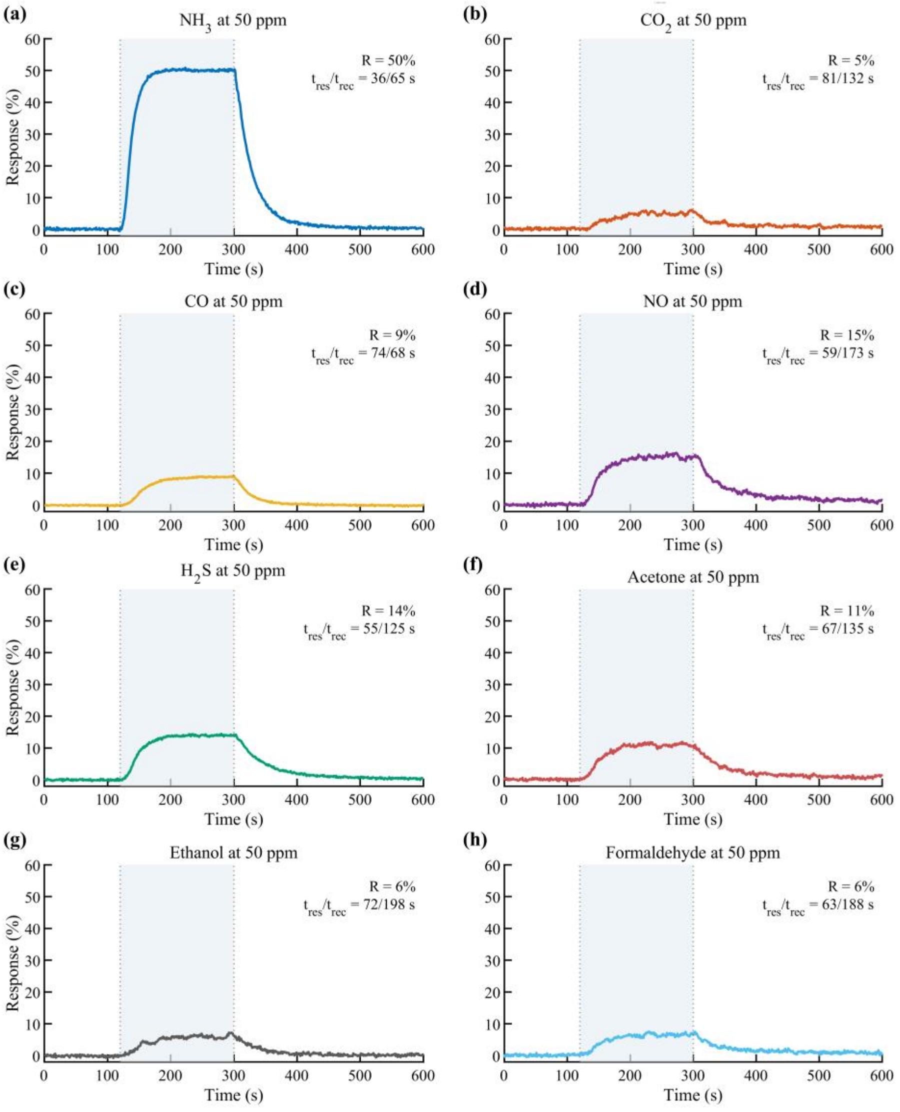

Supplementary Figure S4. Dynamic responses to NH_3, CO_2, CO, NO, H_2S, acetone, ethanol, and formaldehyde at 50 ppm.

S5. COMSOL Simulation and Dataset Construction

The transient evolution of NH_3 was simulated in an 800 × 500 × 400 mm confined chamber using COMSOL Multiphysics. A 10 s heating excitation was applied to promote volatilization and diffusion from a 1% ammonia-water source located at the bottom of the chamber. The COMSOL reference model coupled non-isothermal laminar flow, fluid heat transfer, and diluted-species transport. For the reduced-order PINN constraint, the NH_3-air diffusion coefficient was represented by an effective constant value of $D = 28.0$ $mm^2\ s^{-1}$ ($0.28\ cm^2\ s^{-1}$), while the higher-fidelity COMSOL fields supplied the supervised prior data.

The simulated evolution exhibits three representative stages. First, the released NH_3 accumulates above the source and expands radially under the combined effects of concentration gradients and thermal buoyancy. Second, the rising plume reaches the chamber boundaries and is redirected along the upper and side walls, producing wall-attached spreading and irregular concentration fronts. Finally, the concentration field develops a vertically stratified and progressively homogenized distribution as the initial thermal contribution dissipates and molecular diffusion becomes dominant.

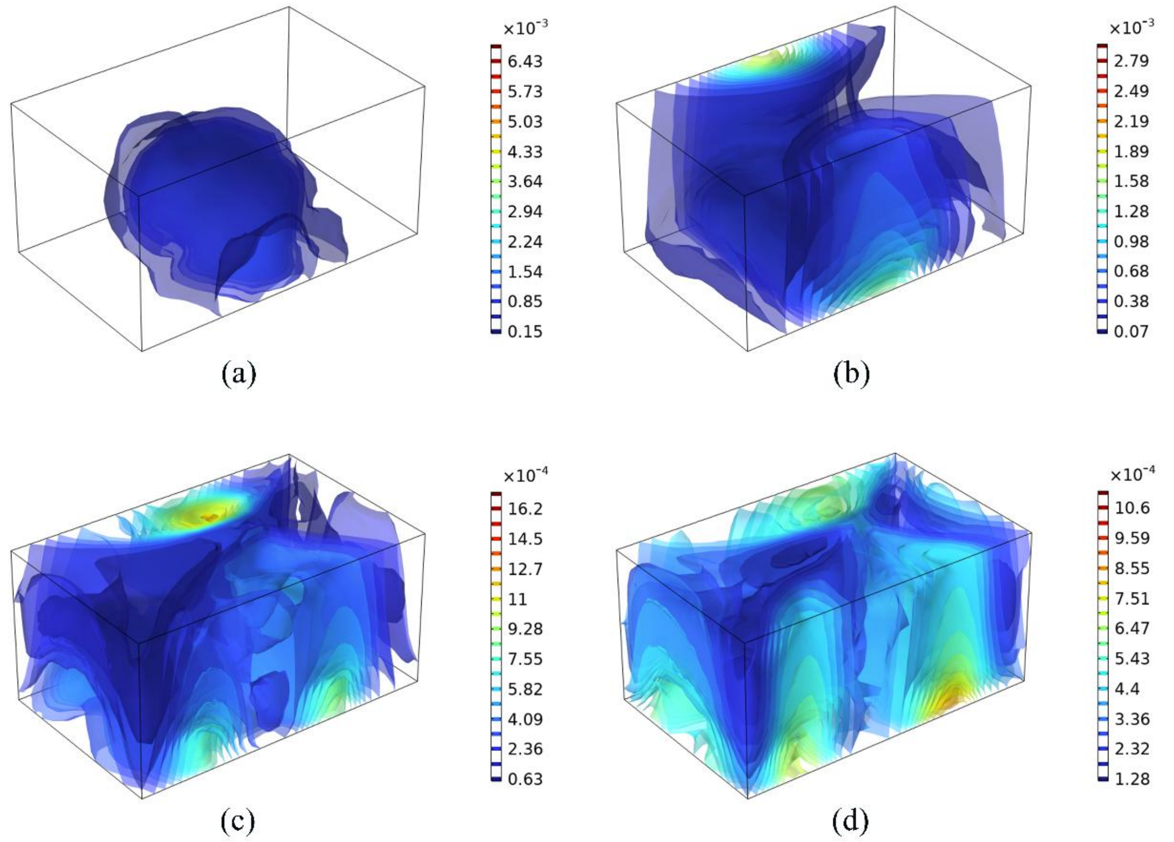

Supplementary Figure S5. COMSOL-simulated spatiotemporal evolution of NH_3 in the confined chamber: (a) initial accumulation; (b, c) boundary contact and wall-attached spreading; and (d) late-stage spatial stratification.

For reproducible offline pre-training, 60,000 supervised concentration points were selected from the COMSOL dataset. At each optimizer update, 6,000 interior collocation points covering the normalized 0–300 s domain and 1,200 points on the six insulated boundaries were resampled to evaluate the PDE and Neumann-boundary losses. The final main model was trained for 500 epochs with a mini-batch size of 8192. This division keeps the high-fidelity simulation data in the offline training stage while reserving the distributed sensing observations for rolling-horizon online correction.

S6. Device-to-Device Reproducibility of the 27-Sensor Array

Twenty-seven PPy/Graphene/WO_3-3 sensor devices were evaluated at 30 ppm NH_3 under the same acquisition procedure. The light-blue traces represent the responses of the individual devices, whereas the dark-blue trace represents their mean response. This comparison directly evaluates device-to-device consistency across all 27 sensing units intended for the multichannel array.

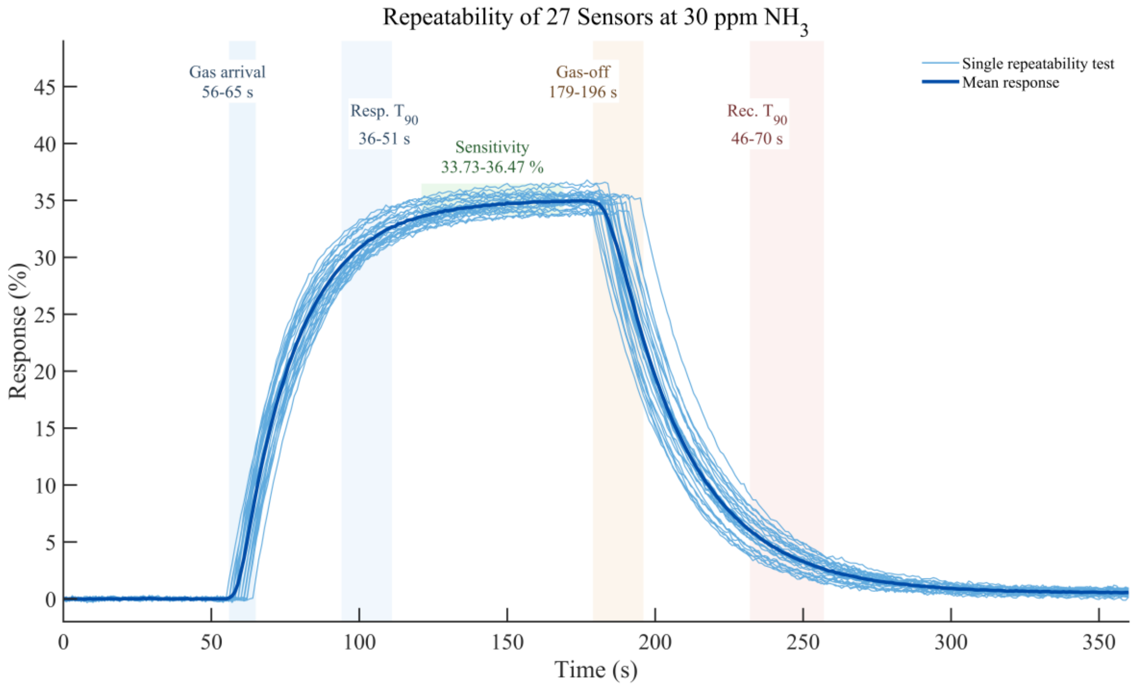

Supplementary Figure S6. Device-to-device reproducibility of 27 PPy/Graphene/WO_3-3 sensors at 30 ppm NH_3. Light-blue lines show individual sensor responses and the dark-blue line shows the mean response; shaded intervals indicate the ranges of gas arrival, T_{90} response, gas-off, and T_{90} recovery times.

All 27 devices exhibited the same response direction and similar rise, plateau, and recovery profiles. Their sensitivities were distributed between 33.73% and 36.47%. The gas-arrival times ranged from 56 to 65 s, the response T_{90} values ranged from 36 to 51 s, the gas-off times ranged from 179 to 196 s, and the recovery T_{90} values ranged from 46 to 70 s. The close grouping of the individual curves and the absence of anomalous response trajectories demonstrate satisfactory device-to-device reproducibility for the 27-sensor array. This result complements the cyclic repeatability and 30-day stability tests reported in the main manuscript.

S7. Real-Time Sensor-Input Validation of Sensor-observation-driven RHDA

The 27 calibrated concentration sequences were experimentally recorded using the fabricated sensor array and streamed to Sensor-observation-driven RHDA at 10 s update intervals. The corresponding COMSOL node concentrations were used only as reference values for assessing input fidelity and were not used as RHDA inputs.

The experimentally measured sensor observations showed high agreement with the corresponding COMSOL node references, with r = 0.987, an overall input RMSE of 0.340 ppm, and an SNR of 20.29 dB. Channel-wise input RMSE values ranged from approximately 0.23 to 0.45 ppm, with no anomalous channel failure. Representative channel 05 captured the overall temporal concentration trend, with only localized positive and negative deviations.

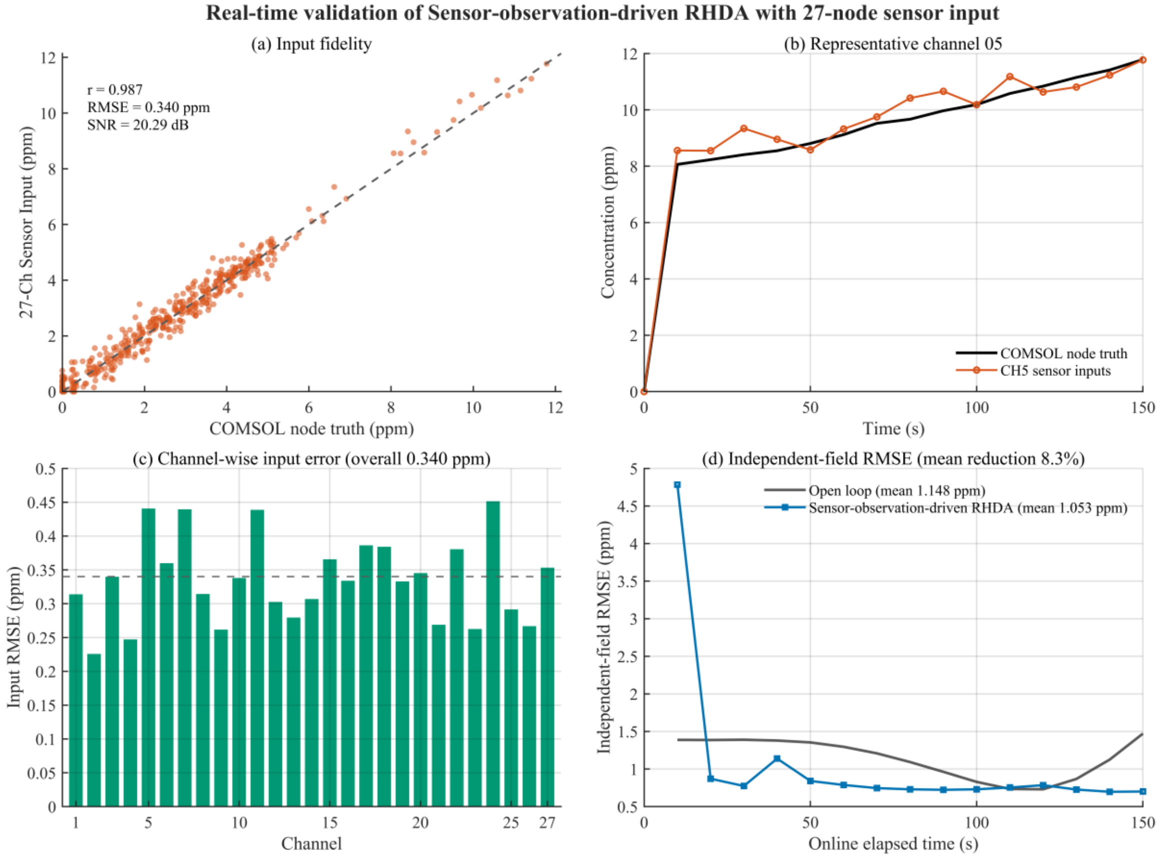

Supplementary Figure S7. (a) Agreement between the 27-Ch sensor input and the COMSOL node references; (b) temporal response of representative channel 05; (c) input RMSE across the 27 channels; (d) comparison of independent-field RMSE between the open-loop PINN and Sensor-observation-driven RHDA.

Across the independent field, the open-loop PINN and Sensor-observation-driven RHDA achieved mean RMSE values of 1.148 and 1.053 ppm, respectively. Sensor-observation-driven RHDA therefore reduced the mean RMSE by 0.095 ppm, corresponding to 8.3%. A large initialization transient occurred at the first update, after which the error rapidly decreased and remained below the open-loop result for most evaluation times. Despite observation noise, Sensor-observation-driven RHDA extracted useful correction information from the streamed 27-node observations.
